# Glutathione Relevance in the Response of Ciliate *Tetrahymena thermophila* Against Metals/Metalloids and Other Environmental Stressors

**DOI:** 10.3390/biology15181616

**Published:** 2026-09-14

**Authors:** Ruth Ortega, Ana M. Martin-González, Juan-Carlos Gutiérrez

**Affiliations:** Department of Genetics, Physiology and Microbiology, Faculty of Biology, Complutense University of Madrid, 28040 Madrid, Spain; rutortega@hotmail.com (R.O.); anamarti@bio.ucm.es (A.M.M.-G.)

**Keywords:** glutathione, metals/metalloids, oxidative stress, glutamate cysteine ligase, glutathione reductase, qRT-PCR, ciliates, *Tetrahymena thermophila*

## Abstract

The tripeptide glutathione (GSH) is the primary and most abundant non-protein thiol compound present in all aerobic organisms. The main function of this molecule is cellular antioxidant defense. For the first time in ciliates, using the eukaryotic microorganism *Tetrahymena thermophila* as a model organism, several parameters (cell mortality, molecules containing −SH groups, and total glutathione) have been evaluated under the toxic effects of various chemical stressors (including several metals/metalloids), either in combination with or without the inhibitory effects of BSO (a GSH biosynthesis inhibitor) or NEM (an inhibitor of molecules containing −SH groups). Oxidative stress caused by many of these stressors is mitigated jointly by GSH and molecules containing −SH groups (such as metallothioneins). Likewise, it requires the over-expression of GR (glutathione reductase) and GCL (glutamate cysteine ligase) genes. Bioinformatic and phylogenetic analysis of these enzymes, involved in the reduction in oxidized glutathione and in its biosynthesis, confirms their correct identification, evolutionary conservation and clustering with the taxonomic group of the ciliate species to which they belong.

## Simple Summary

For the first time in ciliates, the role of glutathione in cellular defense against various chemical stressors has been analyzed using flow cytometry (to evaluate molecules with −SH groups or glutathione) or enzymatic analysis (to assess total glutathione), in a series of experiments that combine the effect of chemical stressor with the presence or absence of inhibitors of either GSH biosynthesis (BSO) or molecules containing −SH groups (NEM). Oxidative stress caused by many of these stressors is mitigated jointly by GSH and molecules containing −SH groups (such as metallothioneins). Similarly, qRT-PCR analysis of genes encoding enzymes involved in biosynthesis (glutamate cysteine ligase) or glutathione-oxidized reduction (glutathione reductase) has shown that these genes need to be over-expressed in response to the toxic effects of these stressors. Both the bioinformatic and phylogenetic analyses of these *T. thermophila* enzymes, as well as their comparison with those from other ciliates, have revealed their high level of evolutionary conservation and their clustering into their respective taxonomic groups.

## 1. Introduction

Glutathione (GSH) is a tripeptide (L-gamma-glutamyl-L-cysteinylglycine), which is the main non-protein thiol compound present in all aerobic organisms, animals [1,2], fungi, plants [3] and bacteria (many Gram-negative and some Gram-positive) [4]. It is found in concentrations ranging from 0.1 to 20 mM, and >98% of it is in reduced form (GSH) compared to the disulfide-oxidized form (GSSG) [5,6]. In eukaryotes, it is abundant (80–85%) in the cell cytosol, 10–15% is in the mitochondria, and a small amount (<10%) is in the endoplasmic reticulum [7,8].

The importance of GSH is mainly due to two structural characteristics: 1—the existence of a gamma-type bond between the glutamic acid and cysteine residues, which protects it from proteases [2], and 2—the free thiol groups of the cysteine residue. As a result of glutathione oxidation, a disulfide bridge forms between two GSH molecules, giving rise to disulfide glutathione (GSSG). GSH/GSSG redox potential is very low (E_0_ = −240 mV), which means that it has a high tendency to oxidize and therefore a high reducing character. Glutathione reductase (GR) is the enzyme (EC 1.6.4.2) responsible for reducing GSSG and thus regenerating GSH to its functional reduced form. It follows that the main function of this molecule is to be involved in cellular antioxidant defense, in addition to other functions, such as the following: (a) Maintaining intracellular redox balance. GSH is involved in the formation and protection of thiol groups in proteins (enzymes) and non-protein molecules such as CoA. It can interact with certain residues in the active center of many enzymes through reactions known as glutathionylation [9], catalyzed by thiol group transferases (such as glutaredoxins) [10,11]. (b) Formation of DNA precursors [12]. (c) Transport and storage of certain amino acids [2]. (d) Detoxification of inorganic (metals/metalloids) and organic xenobiotics (PCBs, PAH, pesticides, dioxins, etc.).

GSH can conjugate, spontaneously or enzymatically (by glutathione transferases) [13], with various electrophilic compounds, including environmental pollutants and cellular metabolism products, making them more soluble, less toxic, and facilitating their expulsion from the cell [14]. Similarly, the −SH group from glutathione has a high affinity for metal cations (Hg(II), Cd(II), As(V), As(III), Pb(II), Zn(II), and Cu(II), among others), chelating these ions, immobilizing them, and preventing them from interacting with essential biological molecules, thus decreasing their toxic effect [15,16]. (e) Detoxification of reactive oxygen species (ROS) and nitrogen species (RNS). Mitochondria are the main source of ROS (hydroxyl anions and hydrogen peroxide, among others), which are highly oxidizing and reactive. H_2_O_2_ can be reduced by catalase or glutathione peroxidase (using GSH as a source of reducing power). In addition, GSH can react directly with various oxygen or nitrogen free radicals [17]. (f) In plants [18], yeasts [19], microalgae [20], fungi [21], nematodes [22], and bacteria (cyanobacteria) [23], GSH is polymerized to oligopeptides called phytochelatins, which bind metals through their numerous thiol groups. In the ciliate *Tetrahymena thermophila*, there is a pseudo-phytochelatin synthase, which appears to be inactive in phytochelatin biosynthesis [24], like what occurs in some cyanobacteria (*Nostoc* sp.), which are also biosynthetically inactive but capable of hydrolyzing GSH [25]. In addition to all these functions, GSH regulates the cell growth cycle and apoptosis, and modulates the immune response and brain metabolism [6,26].

GSH is biosynthesized by a specific non-ribosomal pathway, which occurs in two phases, both of which are ATP-dependent. In the first phase, glutamate and cysteine are joined by a gamma (γ) bond in a process catalyzed by the enzyme glutamate cysteine ligase (GCL) (also known as γ-glutamylcysteine synthase (EC 6.3.2.2)), which is formed by two subunits (heterodimer), the catalytic and the modifying subunit [6]. In bacteria and yeasts, it consists of a single unit with dual functionality (both activities) [27]. During the second step, glycine is added by glutathione synthase (GS) (EC 6.3.2.3), resulting in the tripeptide GSH. GS consists of two identical subunits (homodimer) [28]. In GSH biosynthesis, the enzyme GCL is essential and is inhibited by its end-product (or GSH) [29,30]. This is confirmed by the fact that overexpression of the GS gene does not increase the GSH intracellular amount, but overexpression of the GCL gene does increase it [31]. Among eukaryotes, the protective capacity of GSH against oxidative stress caused by metals and other toxic environmental agents has been extensively analyzed in plants [32,33], animals [34], yeasts, microalgae [35], and some parasitic protists [36,37]. In contrast, little is known about glutathione and its response to environmental stressors in ciliate protozoa.

*Tetrahymena thermophila* is a eukaryotic microorganism widely used as a model organism in ecotoxicological studies, in part because of its similarities to higher animal cells [13,15]. In this study, to determine the relative importance of GSH and other molecules with thiol groups in response to different abiotic stressors in the ciliate model *T. thermophila*, populations of this ciliate were treated or not with inhibitors such as BSO or NEM (2 or 24 h treatments), which block GSH biosynthesis or molecules with −SH groups, respectively, together with different abiotic stressors (metals/metalloids, oxidizing agents, or acidic pH). Thiol groups and cytotoxicity were assessed using flow cytometry. Similarly, total GSH levels were determined enzymatically both throughout cell growth and under stress from the same stressors, together with the presence or absence of inhibitors. In addition, the expression of genes encoding the monomeric enzyme GCL or just one GR, involved in the first phase of GSH biosynthesis or reducing GSSG, respectively, was analyzed under the stress of a wide variety of stressors. All these results provide us with a broad picture of the relevance of GSH and other molecules with thiol groups in cellular defense against various abiotic stressors.

## 2. Materials and Methods

### 2.1. Ciliate Strain, Culture Conditions, Stress Treatments and Inhibitors

*Tetrahymena thermophila* strain SB1969, kindly supplied by Dr. E. Orias (University of California, USA), was cultured in PP210 medium (2% proteose peptone (Pronadisa, Condalab, Madrid, Spain)) supplemented with 10 μM FeCl_3_ and 250 μg/mL of both streptomycin sulfate and penicillin G (Sigma-Aldrich, Merck Life Science S.L.U., Madrid, Spain) for 24 h at 30 ± 1 °C. In the TthGCL gene expression analysis, log-phase 50 mL *T. thermophila* cultures (~2 × 10^5^ cells/mL) were exposed to different stressful conditions: metals/metalloids, such as 27 μM Cd(II) (CdCl_2_), 80 M Cu(II) (CuSO_4_·5H_2_O), 604 μM Pb(II) (Pb(NO_3_)_2_), 30 μM As(V) (Na_2_HAsO_4_·7H_2_O) or 870 μM Zn(II) (ZnSO_4_·7H_2_O) in PP210 medium for 2 or 24 h at 30 °C. These metal concentrations correspond to approximately half the LC_50_ values calculated for *T. thermophila* strain SB1969 as previously reported [38] and resulted in negligible cell mortality. The following organic compounds were used as oxidative stress inducers: the herbicide Paraquat (PQ) (1,1’-dime til-4,4’-bipyridyl dichloride) at 7700 μM in PP210 medium (24 h exposure) [39]; menadione (MD) (2-methyl-1,4-naphthoquinone), with which a 5 M solution in chloroform was prepared and from which a 2000 μM solution in PP210 medium was the final used concentration (2 h exposure) [40]; and CDNB (1-chloro-2,4-dinitrobenzene), with which a 10 mM ethanol solution was prepared and from which a 1/5 dilution was made until a 200 μM concentration in PP210 was obtained (2 h treatment). It is a substrate and inducer of GSTs (glutathione-S-transferases) [41], which causes superoxide anions and oxidative stress [42]. Other abiotic stressors were PP210 medium at basic or acidic pH (pH 9 or pH 5, 24 h exposures). Starvation stress was induced by maintaining the culture (24 h) in 10 mM Tris-HCl buffer (pH 6.8).

For the determination of glutathione and molecules with thiol groups, treatments were carried out in Tris-HCl buffer for 2 or 24 h (30 °C) with the following stress agents: Cd (CdCl_2_) at 1.32, 1.54, or 1.76 µM; As (V) (Na_2_HAsO_4_·7H_2_O) at 33, 66, or 133 μM; PQ at 2.5, 5, or 10 mM (24 h treatments); MD at 5, 7.5, or 10 mM (2 h treatments). These concentrations are considerably lower than those used in PP210 medium cultures, since the organic matter of this growth medium chelates metal cations, requiring a higher concentration to exert a toxic effect [39]. The inhibitors used in conjunction with exposure or non-exposure (control) to the above-mentioned stressors were: BSO (DL-buthionine-sulfoximine) (at 100 μM in Tris-HCl), which is a specific inhibitor of GCL (the enzyme limiting GSH biosynthesis) [43,44], or NEM (N-ethylmaleimide) (50 µM in Tris-HCl), which is a less specific electrophilic agent that interacts with and blocks the −SH groups of cysteine residues from proteins and peptides [45,46]. All previously mentioned chemicals were purchased from Sigma-Aldrich (Merck Life Science S.L.U., Madrid, Spain).

### 2.2. GSH or Thiol Group Determination by Flow Cytometry

To determine the GSH amount by fluorescence, the fluorochrome CellTraker Green CMFDA (5-chloromethylfluorescein diacetate) (Molecular Probes, Fisher Scientific S.L. Alcobendas, Madrid, Spain) was used. Non-fluorescent CMDFA passes through the cell membrane, and once inside, it is cleaved by cytosolic esterases, and the resulting product reacts with GSH via its chloromethyl group. The resulting GSH–fluorochrome conjugate is impermeable and fluorescent (Ex/Em: 492/517 nm). It is highly selective for GSH [47], though it can also react with protein −SH groups. To analyze GSH levels exclusively in the live cell population, the fluorochrome propidium iodide (PI) (Molecular Probes) was used jointly [48]. This double labeling allows for the determination of green fluorescence (due to GSH) in living cells and the mortality percentage (red fluorescence) in the cell population. The fluorochromes were excited with a 488 nm argon laser, and the fluorescence emitted by CMFDA was collected on the FL-1 channel (530/30 nm, green) and on the FL-2 channel (585/42 nm, red) for PI. The analysis was carried out using the FACScalibur (Waters Biosciences, Madrid, Spain) flow cytometer at the UCM’s Flow Cytometry and Fluorescence Microscopy Unit. Fluorescence values were normalized relative to the control in each experiment.

The sample preparation for flow cytometry was as follows: (a) For samples treated with BSO from 100 mL of *T. thermophila* culture in the late exponential phase, cells were collected by centrifugation at 500× *g* (3 min). They were then washed twice in Tris-HCl buffer and resuspended in the same buffer at a concentration of 1–3 × 10^5^ cells/mL. Half of the cell suspension was used as control, and the other half was treated with 100 μM BSO for 20 h at 30 °C. After this incubation time, the corresponding stress agent (metal, oxidant, etc.) was added (at different increasing concentrations), and the thiol groups (selectively GSH) in these samples were quantified at 2 or 24 h. (b) For those treated with NEM, cells were collected in a similar manner to that described above. The cell suspensions in Tris-HCl were divided into two sets of tubes, and both were subjected to increasing concentration of the corresponding stress agent (2 or 24 h of exposure). Subsequently, in one of the sets, the thiol groups were blocked with 50 μM NEM (30 min treatment), and then these groups were labeled with the fluorochromes CMFDA and PI (20 min treatment). In both cases (BSO and NEM), 30 µL of CMFDA (20 µM) and 6 µL of PI (5 µg/mL) were added to each flow cytometry tube containing 600 µL of each sample (controls and treatments).

### 2.3. Enzymatic Measurement of Total Glutathione

To determine whether glutathione concentration was directly proportional to cell number, cell extracts were obtained from cell populations with 5 × 10^5^, 10^6^, 5 × 10^6^, 10^7^, 5 × 10^7^, or 10^8^ cells/mL, and the GSH concentration of all of them was measured. All measurements were linear, and the quantity of 10^6^ cells/mL was chosen for future analyses. The desired cell concentrations were adjusted by counting with a Neubauer chamber. Regardless of the number of cells, potential variations in total glutathione levels were also analyzed throughout the growth curve (see Section 3.2).

Cell extracts from the different samples were obtained by collecting 10^6^ cells/mL by centrifugation at 500× *g* (4 °C, 3 min), washing twice with Tris-HCl buffer. Then, 160 µL of a solution containing HCl (10 mM) and EDTA (4 mM) was added to the cell pellet, and cell lysis was performed by two cycles of freezing in liquid nitrogen and thawing in a 37 °C bath. Once the cells were lysed, 40 µL of a 5% sulfosalicylic acid solution (Sigma-Aldrich, Merck Life Science S.L.U., Madrid, Spain) was added to deproteinize the sample. To remove cellular debris, lysates were centrifuged at 10,000× *g* (4 °C, 10 min). The supernatant obtained was rapidly frozen with liquid nitrogen and stored at −80 °C (for a maximum of ten days) until use.

The enzymatic assay for glutathione assessment was performed using the method described by [49]. This method quantifies total glutathione in both its reduced form (GSH) and oxidized form (GSSG). The assays were performed in 96-well microplates with a final volume of 200 µL/well, each containing: 100 mM potassium phosphate buffer (pH 7), 1 mM EDTA, 48 µM NADPH (Roche Farma, Madrid, Spain), 0.031 mg/mL DTNB (5,5’-dithiobis(2-nitrobenzoic acid) (Ellman’s reagent) (Sigma-Aldrich, Merck Life Science S.L.U., Madrid, Spain), 0.115 units/mL GR (glutathione reductase) (Sigma-Aldrich, Merck Life Science S.L.U., Madrid, Spain), and 20 µL of cell extract or a GSH control (Sigma-Aldrich, Merck Life Science S.L.U., Madrid, Spain) at various concentrations (standard curve). First, 20 µL of the cell extract from each sample, the GR enzyme, and NADPH were added, and microplates were incubated at 37 °C (10 min). Subsequently, DTNB was added and kept at room temperature (5 min), immediately followed by spectrophotometric measurement (405 nm).

### 2.4. RNA Isolation and Quantitative RT-PCR

Total RNA was isolated from control and treated *T. thermophila* cultures (~1–3 × 10^5^ cells/mL) using the commercial RNeasy Mini Kit (Qiagen Iberia S.L., Barcelona, Spain). RNA samples were treated with DNase I (Roche Farma, Madrid, Spain) at 37 °C for 30 min. Subsequently 3 μg of each RNA sample was retrotranscribed to cDNA with the 1st Strand cDNA Synthesis Kit for RT-PCR (AMV) (Roche Farma, Madrid, Spain) by following the manufacturer’s directions. cDNA samples were amplified in duplicate in 96 microtiter plates (Applied Biosystems, Fisher Scientific S.L. Alcobendas, Madrid, Spain). Quantitative RT-PCR was carried out in 20 μL reaction mixtures containing 10 μL of SYBR Green PCR master mix 1× (Takara, Condalab, Madrid, Spain), 5 μL of each primer (0.2 μM), which were designed using the Primer Express v3.0.1 software (Applied Biosystems, Fisher Scientific S.L. Alcobendas, Madrid, Spain), primer sequences are shown in Supplementary Table S1, and 5 μL of a 1/10 cDNA dilution from each sample. The α-tubulin gene (TthATUB) was used as an endogenous control or housekeeping gene.

Samples were amplified in an ABI PRISM^®^ 7900 HT Time PCR System thermal cycler (Fisher Scientific S.L. Alcobendas, Madrid, Spain) using the following thermal cycling protocol: 10 min at 95 °C, followed by 40 cycles of 15 s at 95 °C, 30 s at 50 °C and 20 s at 72 °C, and a final step of 1 min at 95 °C and 1 min at 50 °C. The specificity of each primer pair was confirmed by melting curve analysis. Relative gene expression was quantified according to the delta–delta Ct method [50]. Quantification was carried out relative to the reference gene (α-tubulin) respective to each stress treatment (treated sample or control) by subtracting the cycle threshold (Ct) of the reference gene from the Ct of the corresponding gene. All non-template controls (NTCs) and RT minus control were negative. Amplification efficiency (E) was measured by using 10-fold serial dilution of a positive control PCR template. Efficiency parameters were met for each gene (Table S2).

### 2.5. Statistical and In Silico Analysis

For the statistical analysis of flow cytometry and enzyme assay data, a repeated-measures test was performed in cases where there was no interaction between the two factors to be analyzed (stressor and BSO or NEM inhibitor). In cases where the interaction was significant, Student’s t-test was performed for each stress inducer to analyze the inhibitory effect of BSO or NEM. To analyze the effect of the stress inducer, a repeated-measures test was performed separately for each inhibitor. A significant effect was considered when the *p*-value was ≤0.05. All these analyses were performed using the SPSS v.15.0 computer program. For a better understanding of the tables on the statistical analyses of the thiol group (GSH) determination, total glutathione and cell mortality, an example is explained in a text included in the Supplementary Material (Text S1). Gene expression differences were tested for statistical significance by one-way ANOVA followed by Dunnett’s multiple-comparisons test performed with GraphPad Prism 11.0.0 (build 84) (graphpad.com). The *p*-value was fixed at ≤0.05 for statistically significant values.

Sequence alignments and their percent identities were obtained from the BioEdit Sequence Alignment Editor program [51]. Phylogenetic analysis was carried out using the web server NGPhylogeny.fr (https://ngphylogeny.fr/) (accessed on 27 April 2026), and using the programs MAFFT for multiple alignment, BMGE for alignment curation, PhyML (software based on the maximum likelihood) for tree inference with Smart Model Selection, and Newick display (to display a phylogenetic tree as SVG) [52]. The amino acid sequences of the enzymes analyzed (GR, GCL, or GS) were obtained from various databases containing sequenced ciliate genomes, such as the “Tetrahymena Comparative Genome Database” (http://ciliates.ihb.ac.cn/tcgd/) (accessed on 20 April 2026) or “Genome Databases for the Ciliate Research Community” (https://ciliates.org/) (accessed on 17 April 2026).

## 3. Results

### 3.1. Flow Cytometry Analysis

To determine the relative relevance of molecules with free thiol groups (−SH) in the cellular response to a variety of cytotoxic compounds, both cytotoxicity and total thiol groups were quantified. Two types of populations were used, treated with the inhibitors BSO or NEM (2 or 24 h exposures), which reduce GSH levels and block thiol groups, respectively. These *T. thermophila* populations (treated or not (control) with the above inhibitors) were subjected to different environmental stress conditions: oxidative stress caused by Cd(II) or As(V), oxidizing agents (PQ, MD), or acidic pH.

**Figure 1:**
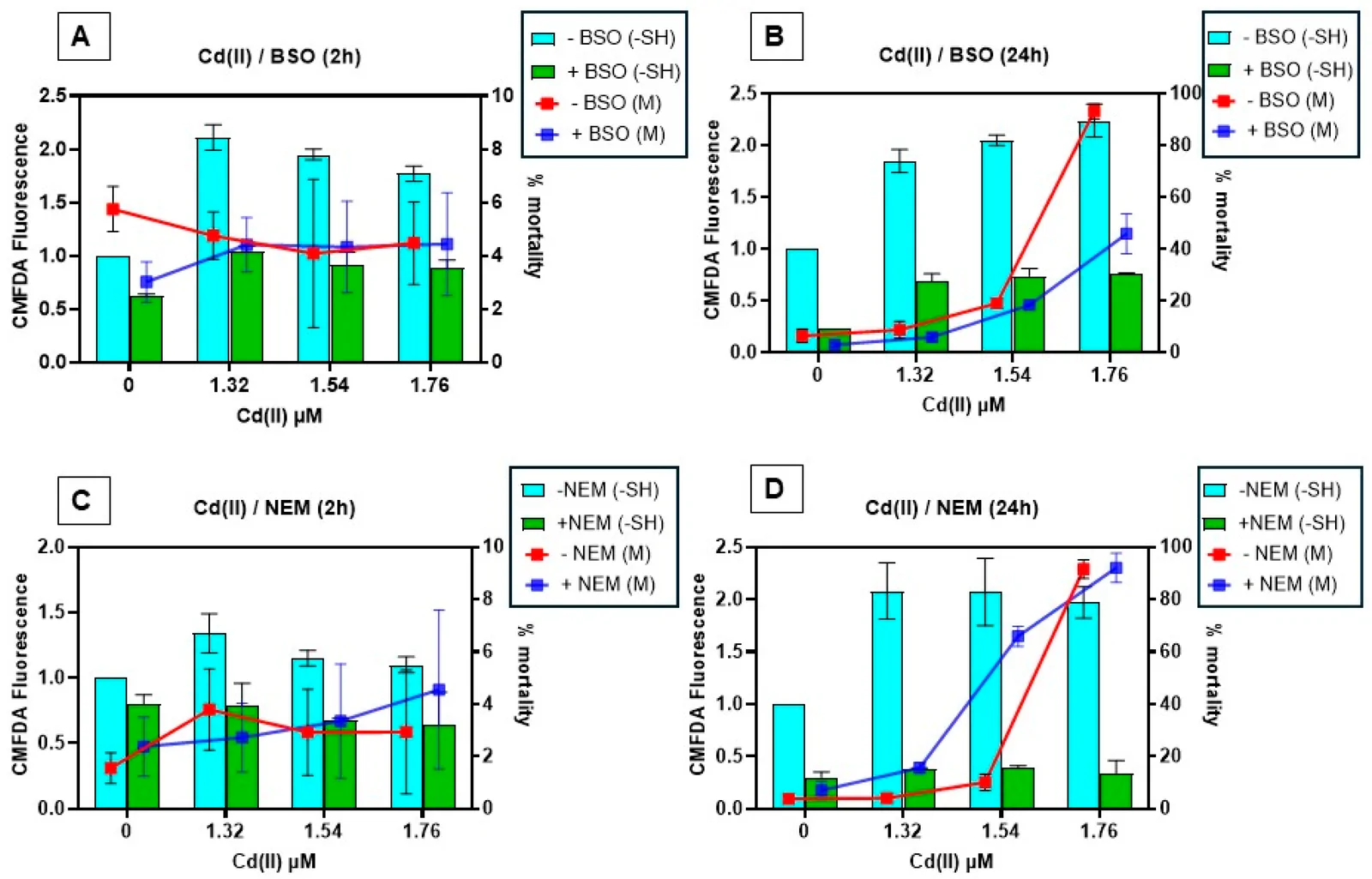
Histograms (bars) showing the number of thiol groups (−SH) (CMFDA fluorescence) (left axis) and the percentage (lines) of cell death (M) (right axis) in *T. thermophila* populations treated (2 or 24 h) with Cd(II), and in the presence or absence of the inhibitors BSO (**A**,**B**) or NEM (**C**,**D**). Table S3A–D show the statistical analysis of these results.

**Figure 2:**
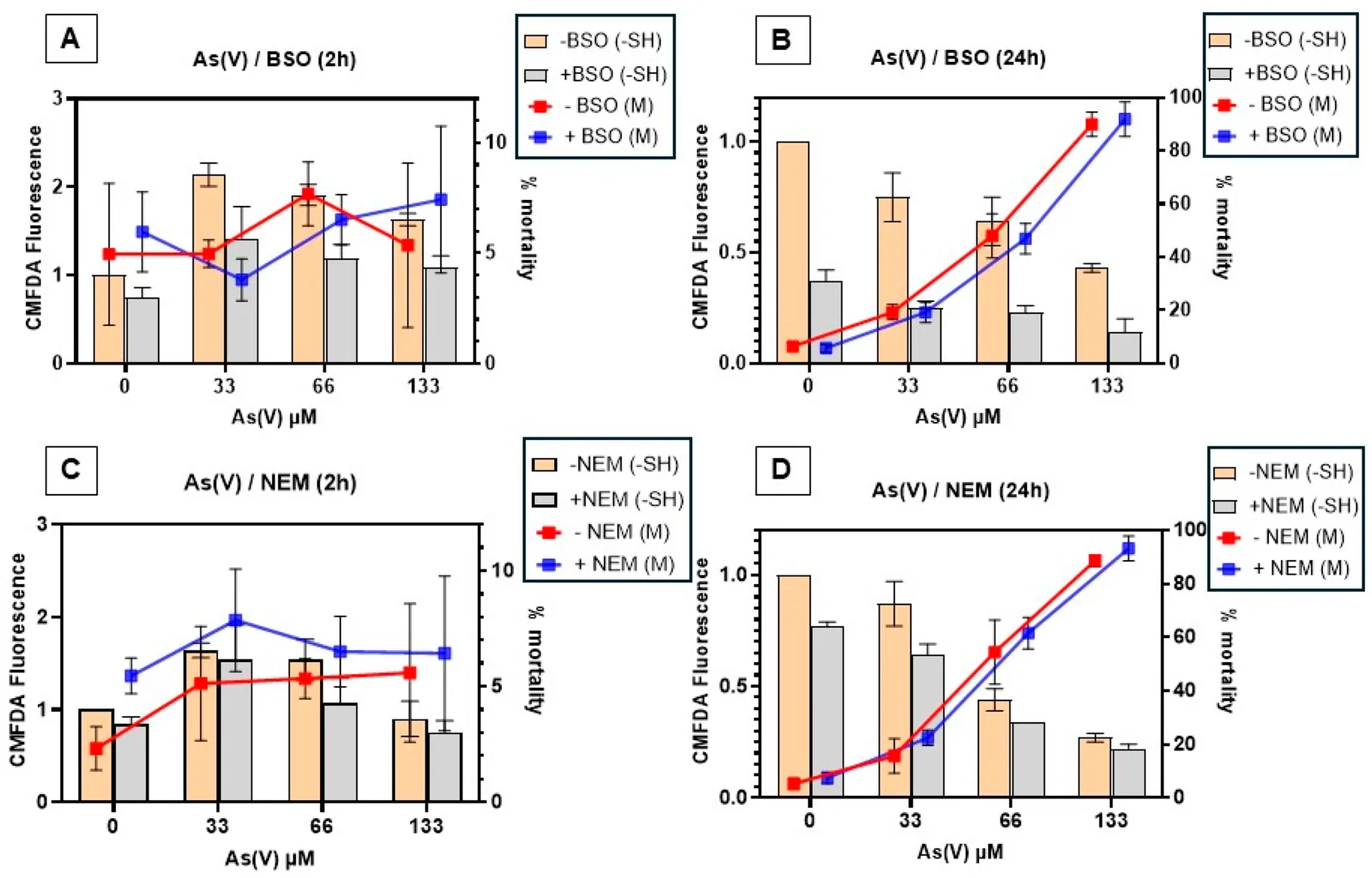
Histograms (bars) showing the number of thiol groups (−SH) (CMFDA fluorescence) (left axis) and the percentage (lines) of cell death (M) (right axis) in *T. thermophila* populations treated (2 or 24 h) with As(V), and in the presence or absence of the inhibitors BSO (**A**,**B**) or NEM (**C**,**D**). Table S3E–H show the statistical analysis of these results.

**Figure 3:**
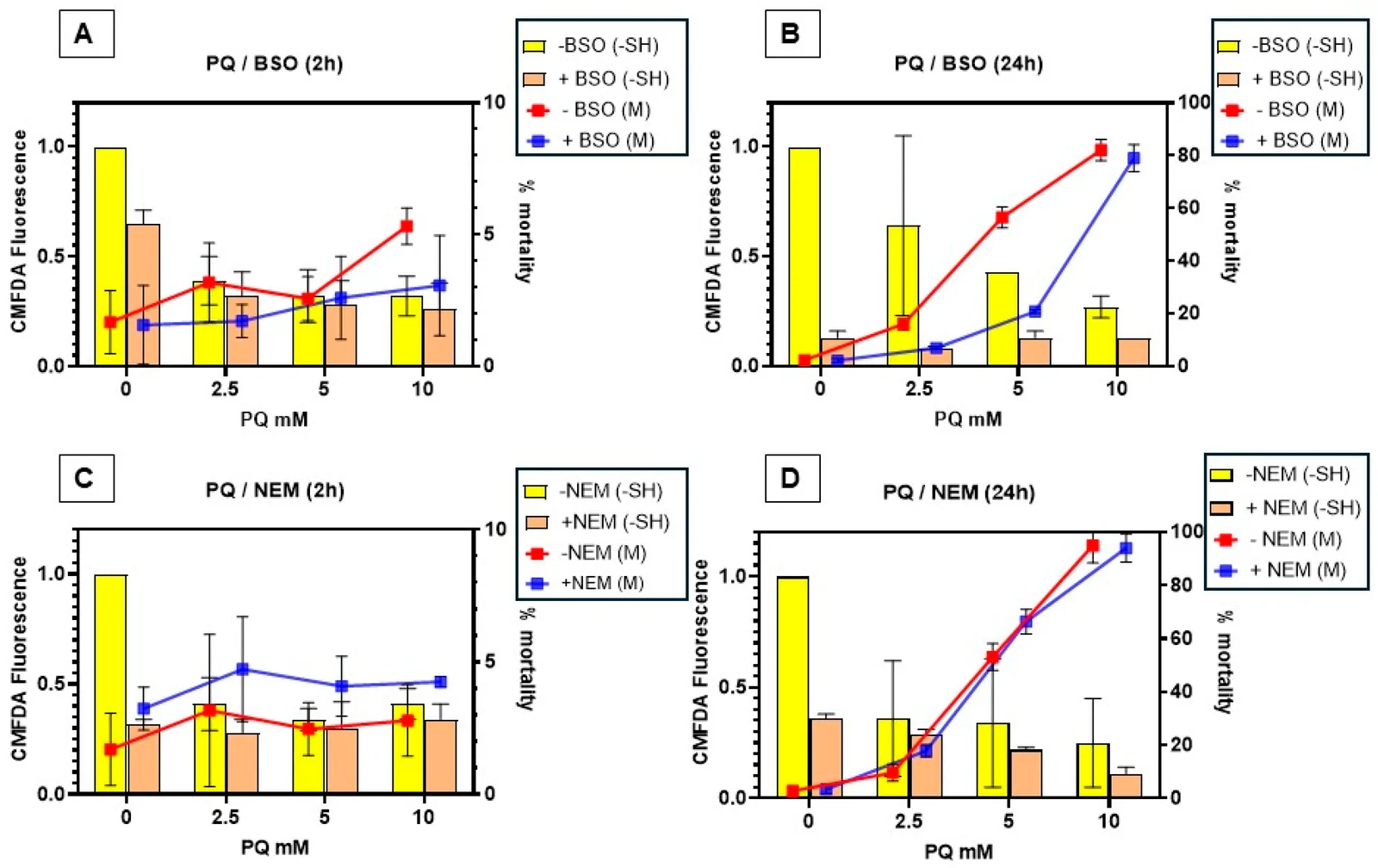
Histograms (bars) showing the number of thiol groups (−SH) (CMFDA fluorescence) (left axis) and the percentage (lines) of cell death (M) (right axis) in *T. thermophila* populations treated (2 or 24 h) with PQ, and in the presence or absence of the inhibitors BSO (**A**,**B**) or NEM (**C**,**D**). Table S3I–L show the statistical analysis of these results.

**Figure 4:**
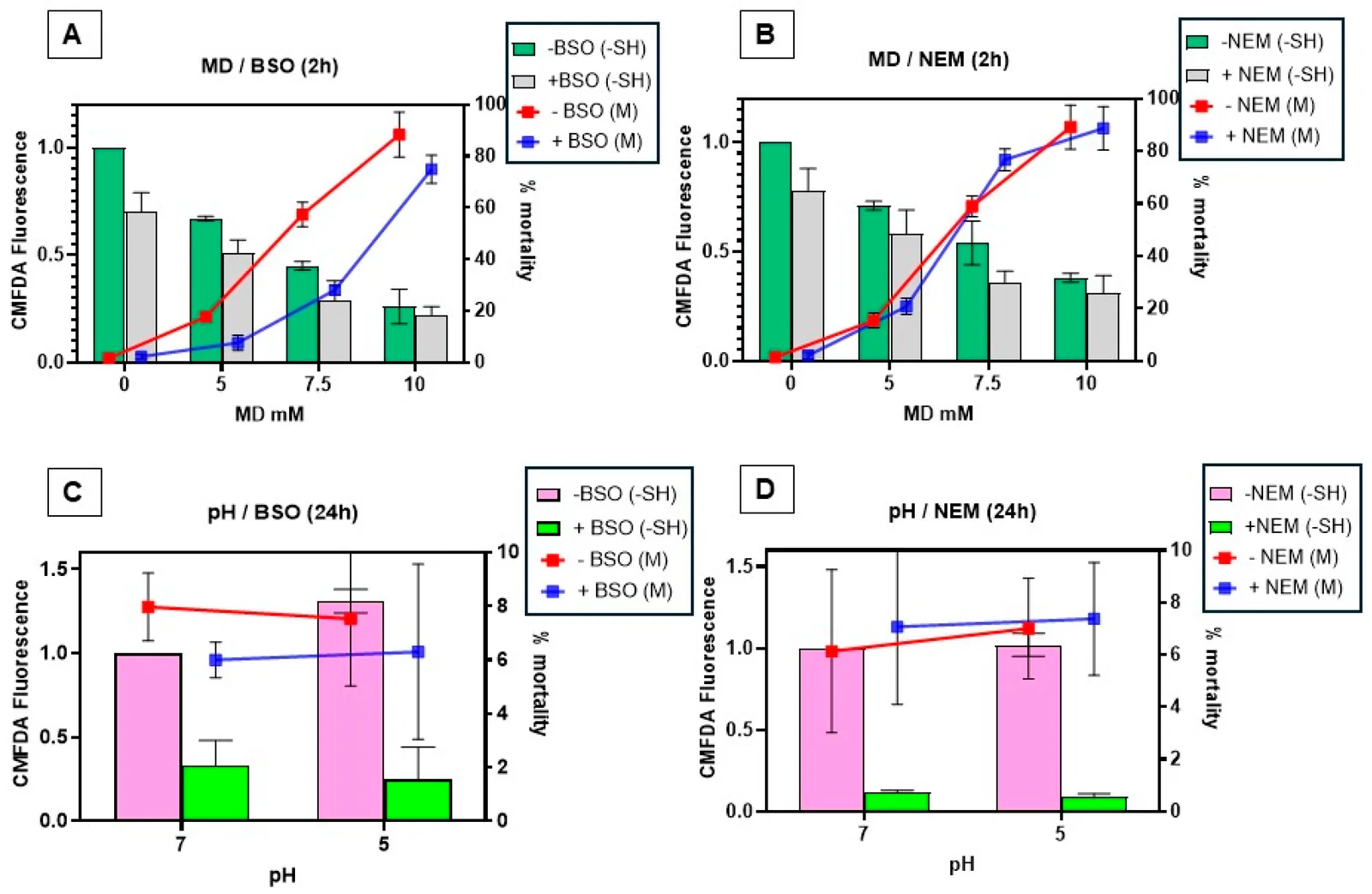
Histograms (bars) showing the number of thiol groups (CMFDA fluorescence) (left axis) and the percentage (lines) of cell death (right axis) in *T. thermophila* populations treated (2 or 24 h) with MD or pH 5, and in the presence or absence of the inhibitors BSO (**A**,**C**) or NEM (**B**,**D**). Table S3M–P show the statistical analysis of these results.

Simultaneous assessment of groups (−SH) and cytotoxicity (cell mortality, M) was performed by flow cytometry, using the fluorochromes CMFDA and PI. The results are shown in Figure 1, Figure 2, Figure 3 and Figure 4, and a Supplementary Table (Table S3) is included for each of these graphs, showing the average values and standard deviations from both the relative fluorescence and mortality percentages for each sample (+BSO, −BSO, +NEM, or −NEM) and for each stress treatment. Similarly, two other attached tables (Table S3A–P) show the results of the statistical analysis for each sample and treatment, indicating the *p*-value for each analysis. Values of *p* ≤ 0.05 were considered significant.

#### 3.1.1. Cd(II) Treatments

Short exposures (2 h) to the three Cd(II) concentrations used cause quite low mortality (Figure 1A,C); however, in longer exposures (24 h) (Figure 1B,D), the mortality rate increases considerably at Cd(II) concentrations of 1.54 or 1.76 μM, and at this last concentration, cell mortality reaches almost 100% of the population (without BSO and with/without NEM), while at this same Cd(II) concentration in the presence of BSO, [EE2.1] mortality decreases significantly (to 50%) (Figure 1B). On the contrary, in the presence of NEM, there is a drastic increase in the percentage of dead cells (70%) at 1.54 µM Cd(II) (Figure 1D), compared to the population with the same Cd(II) concentration but without NEM. At 1.76 µM Cd(II), both populations (with or without NEM) show a mortality rate of almost 100% (Figure 1D).

The presence of Cd(II) also causes significant variations in cellular levels of molecules with (−SH) groups, regardless of exposure time (2 or 24 h) (Figure 1A,D). Cd(II) causes an increase in fluorescence corresponding to an increase, with regard to control, in molecules with (−SH) groups, probably and mainly GSH and metallothioneins (MTs). As expected, the presence of BSO or NEM significantly decreases fluorescence emission, and this difference is greater in populations treated for 24 h (Figure 1B,D). The thiol group value patterns obtained at different Cd(II) concentrations (2 h treatment) (Figure 1A,C) are quite similar, regardless of the BSO or NEM treatments, and something similar occurs with the patterns obtained at 24 h with Cd(II) (Figure 1B,D). At 2 h, the maximum fluorescence value is shown in the samples treated with 1.32 µM Cd(II), in both those with BSO or with NEM (Figure 1A,C). At higher concentrations, fluorescence decreases in both controls and samples treated with BSO or NEM (although in some cases there is no significant difference compared to the sample with lower Cd(II) concentration). In the patterns obtained after 24 h of Cd(II) treatment, the fluorescence values increase with metal concentration or remain similar to those obtained at the lowest concentration. Similarly, treatments with BSO or NEM drastically decrease the fluorescence levels in these samples (Figure 1B,D).

#### 3.1.2. As(V) Treatments

The cytotoxic effect of As(V) is somewhat different from that observed in Cd(II) (Figure 2). However, like Cd(II), mortality at 2 h As(V) treatment is very low, in both the presence or absence of BSO or NEM (Figure 2A,C). After 24 h of treatment, cell mortality is directly proportional to the As(V) concentration (Figure 2B,D), reaching almost 100% mortality at 133 µM of As(V), and this occurs in both the presence or absence of BSO or NEM.

Regarding thiol groups, in short treatments (2 h) and at 33 µM, the lowest concentration of As(V), there is a clear increase in the intracellular concentration of free (−SH) groups, almost double that of the control (without arsenate) (Figure 2A,C). As the concentration of As(V) increases, the levels of free (−SH) decrease, both with and without BSO or NEM. Samples with As(V) without NEM (2 h) show a smaller decrease in (−SH) groups (an average difference of 0.24) (Figure 2C) compared to those with Cd(II) and without NEM (2 h) (a difference of 0.49) (Figure 1C). Twenty-four-hour treatments (Figure 2B,D) show a similar pattern of gradual decrease in the number of (−SH) groups as As(V) increases, inversely correlated with mortality, and this occurs in samples with or without BSO or NEM. This decrease is greater in samples with BSO than in those not exposed to BSO (an average difference of 0.4) (Figure 2B), and greater than the decrease detected (a difference of 0.12) in samples treated with NEM compared to the control (without NEM) (Figure 2D).

#### 3.1.3. PQ Treatments

PQ has little toxic effect on T. thermophila during short exposures (2 h) (Figure 3A,C). However, toxicity increases significantly during prolonged treatments (24 h) (Figure 3B,D). The mortality rate is higher in cell populations not subjected to inhibition of GSH biosynthesis by BSO (at 5 mM PQ, 24 h) compared to those treated with BSO (Figure 3B). However, in those treated with NEM, no significant difference in mortality is observed compared to those without NEM (Figure 3D). Regardless of the concentration and exposure time to PQ, its presence causes a drastic decrease in cellular levels of −SH groups, which are always lower than those from the control (without PQ) (Figure 3C,D). PQ causes such a marked decrease in free cellular thiols that its presence interferes with the inhibitors used (BSO and NEM). However, cells treated with BSO or NEM exhibit lower thiol levels than those observed in cells not treated with the inhibitors (Figure 3C,D).

#### 3.1.4. MD Treatments

MD is quite toxic to T. thermophila, as mortality was 100% at all tested concentrations (5–10 mM) over a 24 h exposure period; therefore, exposure to this oxidant was limited to 2 h for all assays. The results (Figure 4A,B) show a directly proportional relationship between cell death and MD concentration. The presence of BSO reduces the cytotoxicity of MD, particularly at lower concentrations (5 and 7.5 mM MD) (Figure 4A). In contrast, treatments with NEM always result in a slight increase in mortality at the same MD concentrations (Figure 4B). On the other hand, both NEM and BSO reduce levels of free thiol groups at all tested MD concentrations, with similar differences (an average of 0.12). This value (0.12) is lower than that of the MD-untreated group (0.26 average).

#### 3.1.5. Acid pH Treatment

A decrease to pH 5 does not result in a significant increase in cell mortality, regardless of whether the cells are in the presence of the inhibitors BSO or NEM. Regardless of whether the pH is neutral or acidic, treatment with BSO or NEM significantly reduces the number of free thiol groups compared to the controls (Figure 4 panels C,D).

### 3.2. Enzymatic Values of Total Glutathione (GSH/GSSG) Levels Throughout the Growth Curve and Exposed to Various Stressors

Using the CMFDA fluorophore and flow cytometry, it is only possible to quantify total free thiol groups, and differentiation between GSH and other molecules containing −SH groups is not possible. To assess the importance of this tripeptide in response to various stressors, total glutathione levels [reduced form (GSH) + oxidized form (GSSG)] were analyzed enzymatically, as the protocol used only allows for this type of measurement. Previously, to verify whether there are significant variations along the T. thermophila standard growth curve, this analysis was performed in PP210 growth medium at 30 ± 1 °C.

**Figure 5:**
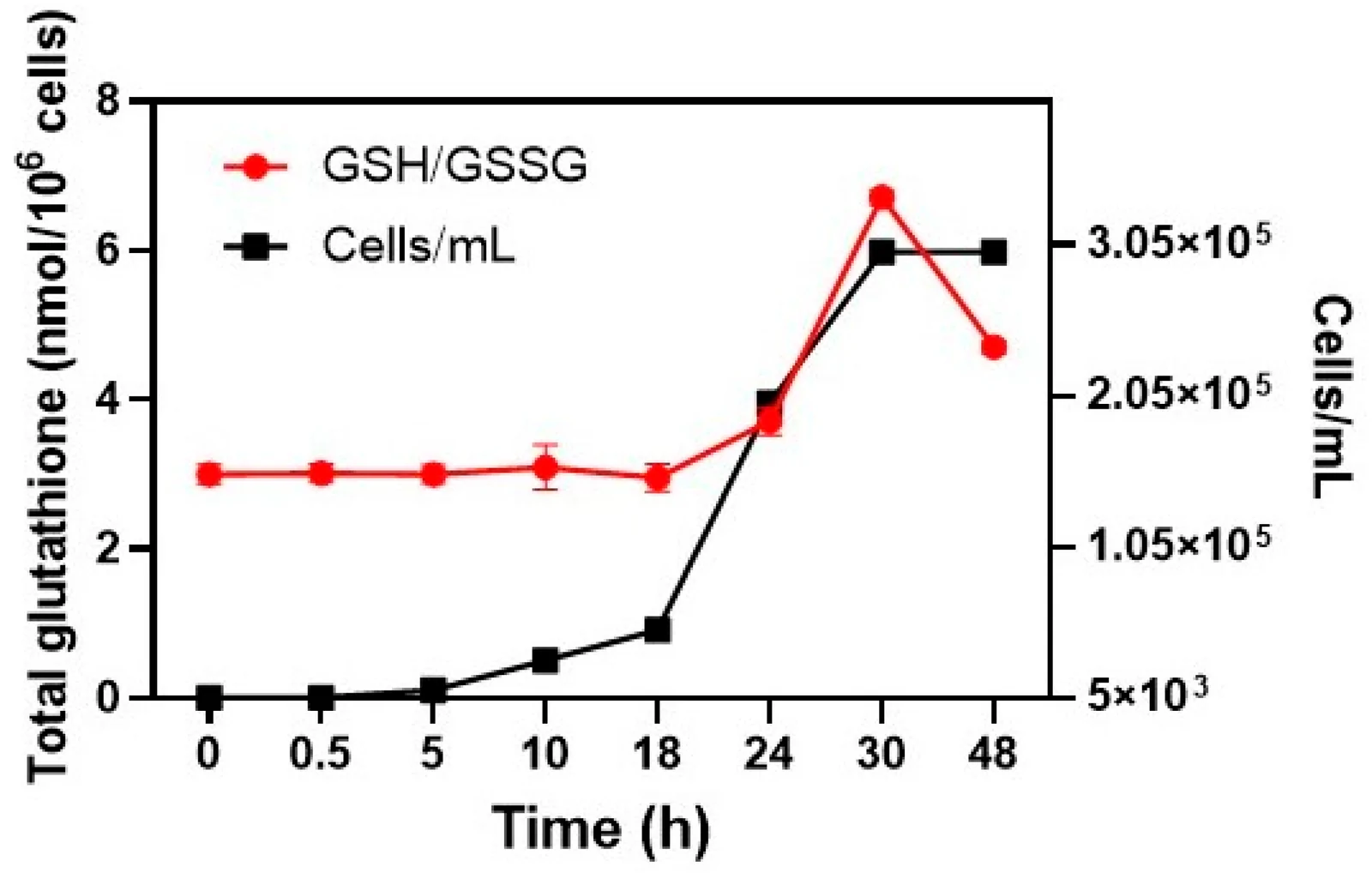
Enzymatic determination of total glutathione levels throughout the *T. thermophila* growth curve. See Table S4 for the average values (samples = 3).

Results are shown in Figure 5 and Table S4. The following growth parameters are obtained from the exponential phase of the growth curve: number of generations (n) = 2.6, generation time (tg) = 4.6 h, and growth rate (μ) = 0.21 h^−1^. Total glutathione levels (expressed as nmol/10^6^ cells) remain constant (around 3 nmol) throughout the latency phase and double during the exponential phase, reaching a maximum value at the end of this phase (Figure 5, Table S4). During the stationary phase, total glutathione levels decrease by approximately 2 nmol from the maximum value reached. Due to these variations in basal glutathione levels throughout the growth cycle, all bioassays (control and treated samples) should be performed using cell populations that are at approximately the same growth stage. Cultures were selected after 24 h, when they had reached approximately the midpoint of the exponential growth phase.

#### 3.2.1. Cd(II) Treatments

**Figure 6:**
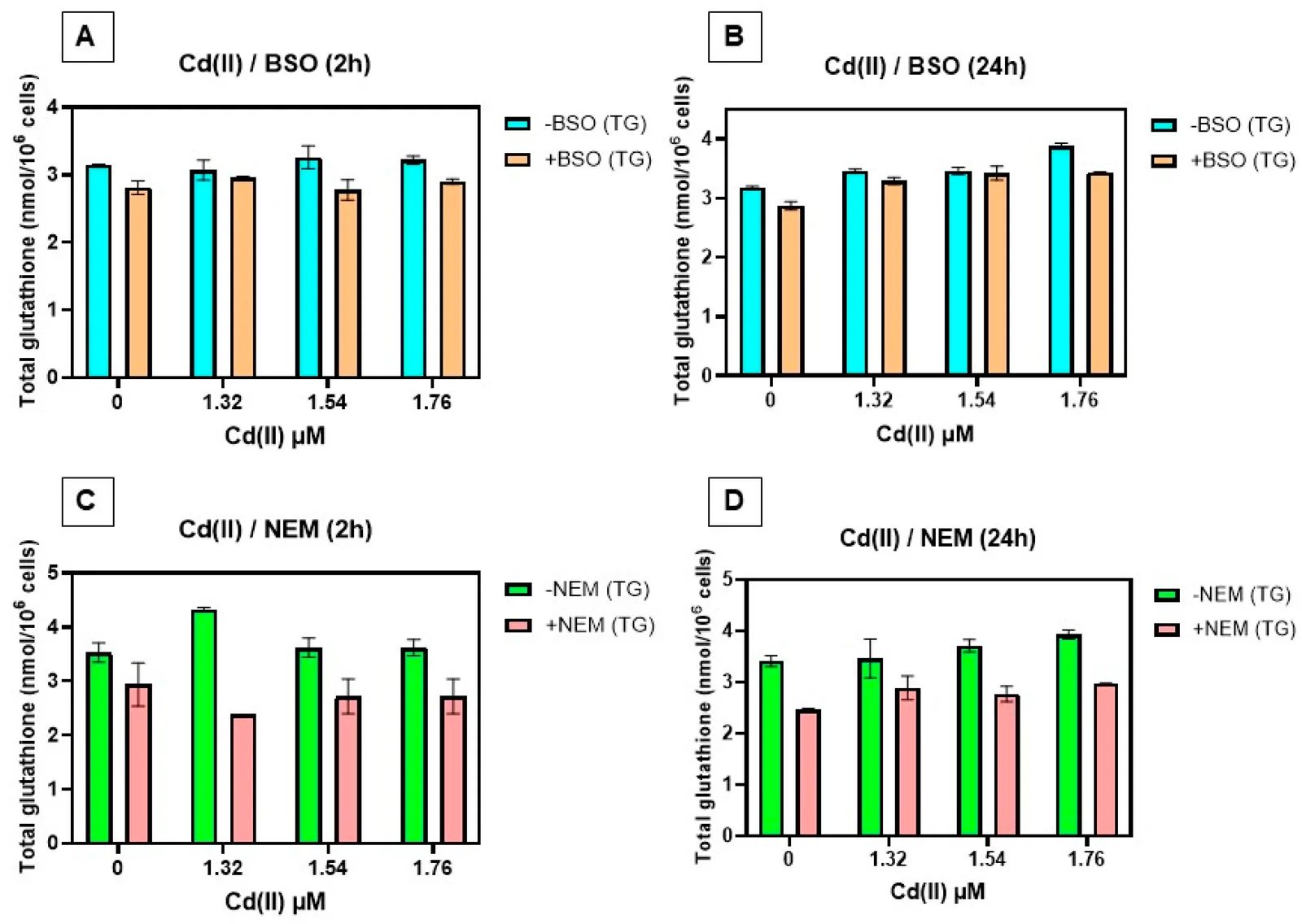
Histograms of total glutathione (TG) levels, determined enzymatically in *T. thermophila* populations treated (2 or 24 h) with Cd(II), and in the presence or absence of the inhibitors BSO (**A**,**B**) or NEM (**C**,**D**). Table S5A–D show the statistical analysis of these results.

*T. thermophila* populations treated for 2 h under different concentrations of Cd(II) show no significant changes in total glutathione content (Figure 6, Table S5). The addition of the BSO inhibitor causes a small but significant decrease in total glutathione, regardless of the concentration of Cd(II) used (Figure 6A). Longer exposures (24 h) to Cd(II) induce a significant increase in total glutathione, which is more apparent at the highest concentration (Figure 6B, Table S5B). The presence of BSO generally causes a small but significant decrease in total glutathione (Figure 6B). Statistical analysis indicates that there is an interaction between Cd(II) and BSO in samples treated for 24 h, although not at 2 h (Table S5B).

Regarding treatments (2 h) with NEM (Figure 6C, Table S5C), it appears to act as a modulator of the cadmium cellular effect. However, statistical analysis does not indicate an interaction between Cd(II) and NEM when exposure is prolonged (24 h) (Figure 6D, Table S5D). In the presence of NEM, there is a significant reduction in total glutathione in all those treated with Cd(II) (2 h) compared to those not treated with NEM (Figure 6C). In contrast, during prolonged exposure (24 h), a slight increase in total glutathione is observed relative to the Cd(II) concentration (Figure 6D). NEM reduces glutathione levels in all cases. It should be noted that the total glutathione reduction caused by NEM (24 h treatment) is slightly greater (mean value of 2.77 nmol) than that caused by BSO (24 h), which has a mean value of 3.25 nmol.

#### 3.2.2. As(V) Treatments

**Figure 7:**
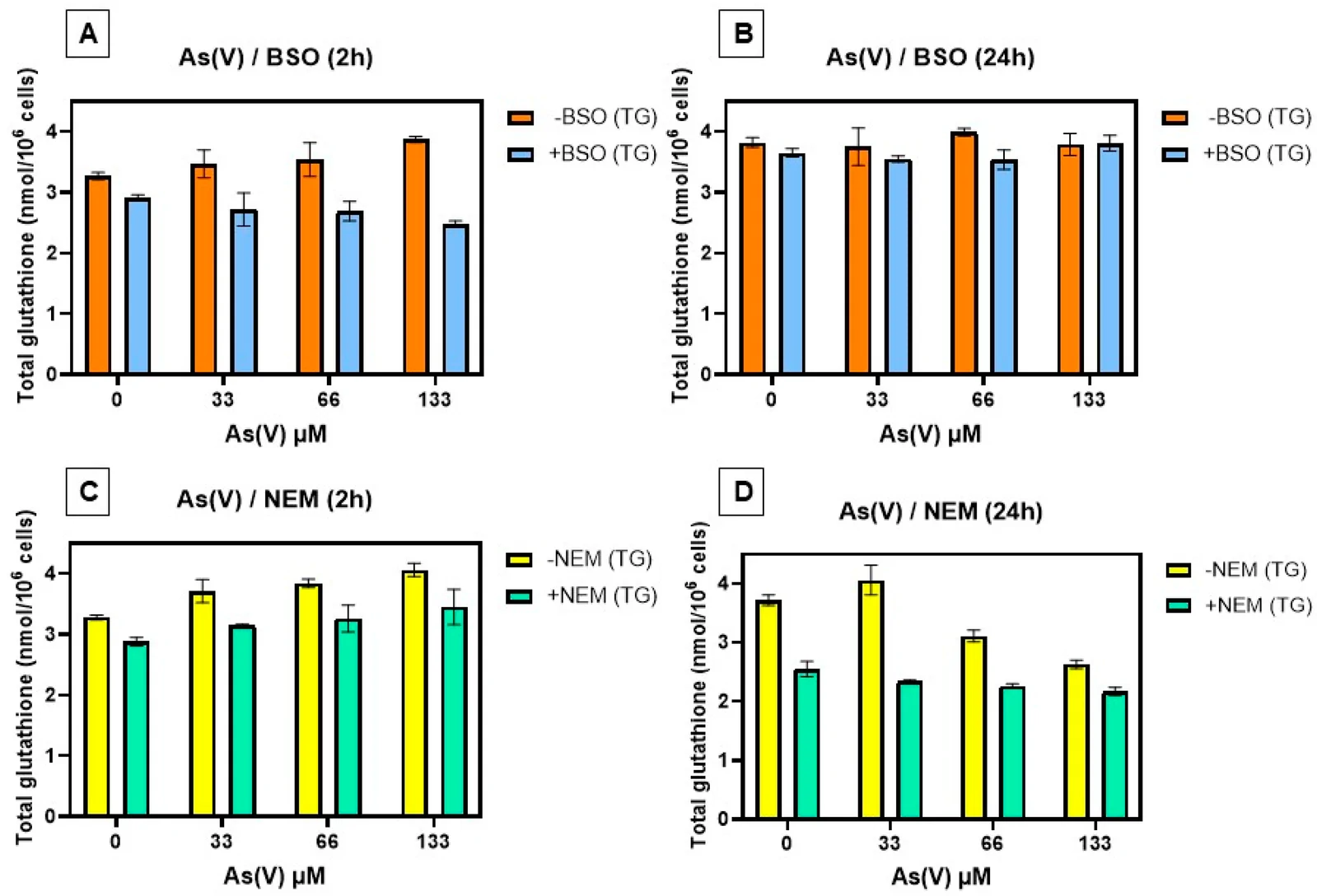
Histograms of total glutathione (TG) levels, determined enzymatically in *T. thermophila* populations treated (2 or 24 h) with As(V), and in the presence or absence of the inhibitors BSO (**A**,**B**) or NEM (**C**,**D**). Table S5E–H show the statistical analysis of these results.

In the presence of As(V), total glutathione levels (without BSO treatment) are very similar across different concentrations of this metalloid, both at 2 and 24 h (Figure 7A,B, Table S5E,F). When BSO is added, slight decreases in total glutathione levels are observed compared to samples without BSO; this decrease (2.7 nmol on average) is more pronounced in samples treated for 2 h than in those treated for 24 h (3.6 nmol on average) (Figure 7A,B). Statistical analysis reveals a significant interaction between As(V) and BSO only when exposure to both elements is brief (2 h). However, in treatments with NEM (Figure 7C,D, Table S5G,H), exposure time appears to be a relevant factor. Thus, a slight increase in total glutathione levels is observed in populations exposed to shorter periods (2 h), and a decrease in these levels when cells were treated for 24 h with both elements (As(V) and NEM). Like Cd(II), the effect of NEM is greater than that of BSO in terms of glutathione reduction.

#### 3.2.3. PQ Treatments

**Figure 8:**
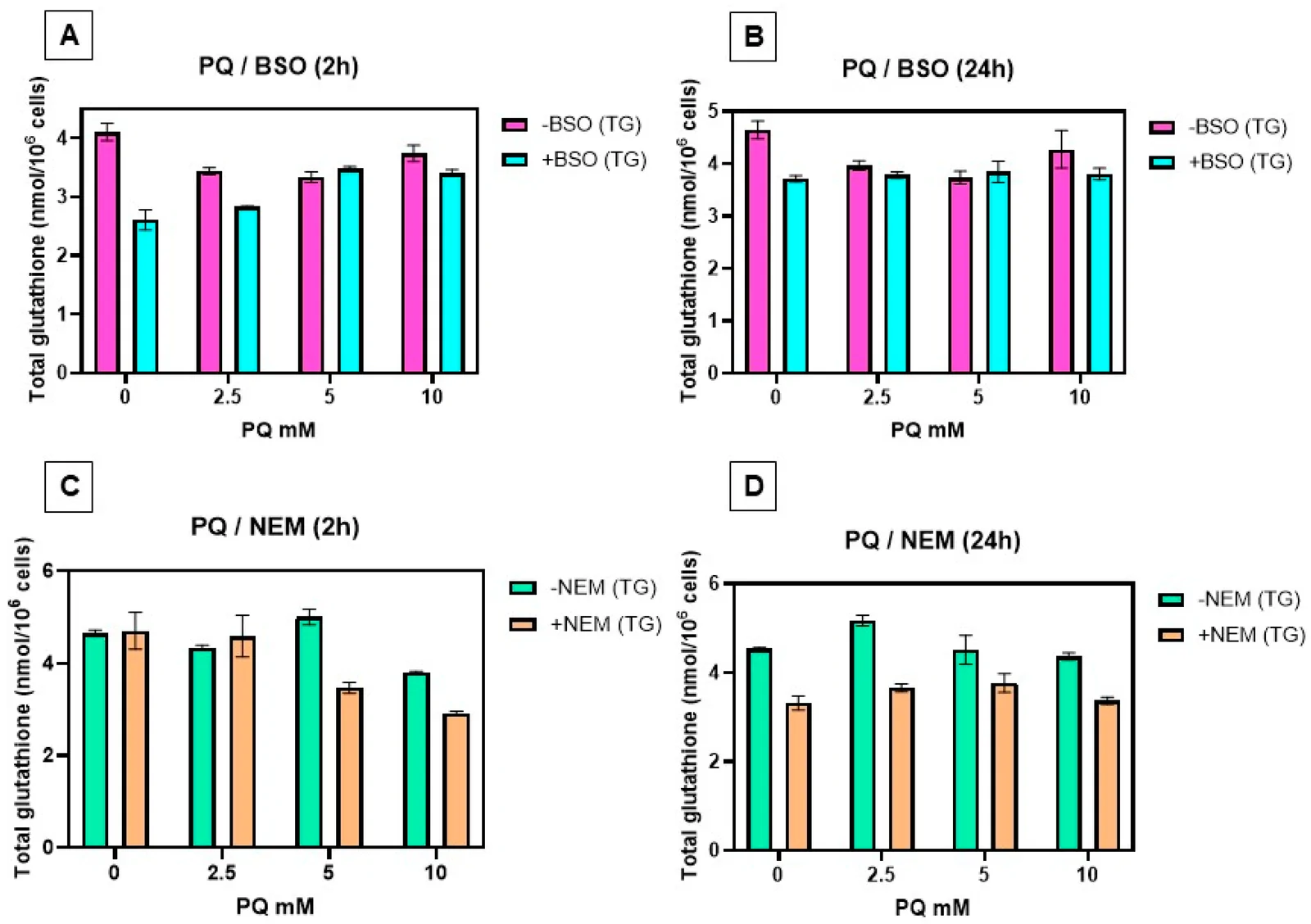
Histograms of total glutathione (TG) levels, determined enzymatically in *T. thermophila* populations treated (2 or 24 h) with PQ, and in the presence or absence of the inhibitors BSO (**A**,**B**) or NEM (**C**,**D**). Table S5I–L show the statistical analysis of these results.

In all cases, treatment with PQ causes slight variations in total glutathione content, regardless of the exposure period and herbicide concentration. For both BSO and NEM, there is a significant interaction between BSO or NEM and PQ during short exposures (2 h), but not during prolonged exposures (24 h) (Figure 8A,B, Table S5I,J). In samples treated with PQ + BSO, an increase in total glutathione levels is observed between the untreated controls and those treated with PQ. In short-term exposures (2 h), there is a significant interaction between PQ and BSO; thus, depending on the presence or absence of BSO, there is an increase or decrease in glutathione levels (Figure 8A). For example, in the absence of BSO, there is a significant decrease in glutathione levels between the control sample and those treated with PQ, whereas in the presence of BSO, there is a significant increase at PQ concentrations ranging from 2.5 to 5 mM (Figure 8A, Table S5I).

In NEM experiments, treatment duration also appears to be an important factor in determining whether there is a significant interaction between NEM and PQ. In short treatments (2 h), there is a significant interaction between PQ and NEM. In NEM-free conditions and after 2 h of treatment with PQ, glutathione levels decrease between the control and the lowest concentration (2.5 mM PQ). However, when the concentration is increased to 5 mM, glutathione levels rise significantly and then decrease again when the PQ concentration reaches 10 mM (Figure 8C, Table S5K). After 24 h exposure and in the presence of NEM, a decrease in glutathione levels is observed compared to those obtained in the absence of NEM, regardless of the concentration of PQ used (Figure 8D, Table S5L). Similarly, 24 h after treatment (as is observed in the 2 h treatment) a pattern of rising and falling glutathione levels is observed. There is a significant increase in glutathione levels between the control and the 2.5 mM PQ-treated sample, but when the concentration is increased to 5 mM PQ, glutathione levels decrease significantly (Figure 8D).

#### 3.2.4. MD Treatments

**Figure 9:**
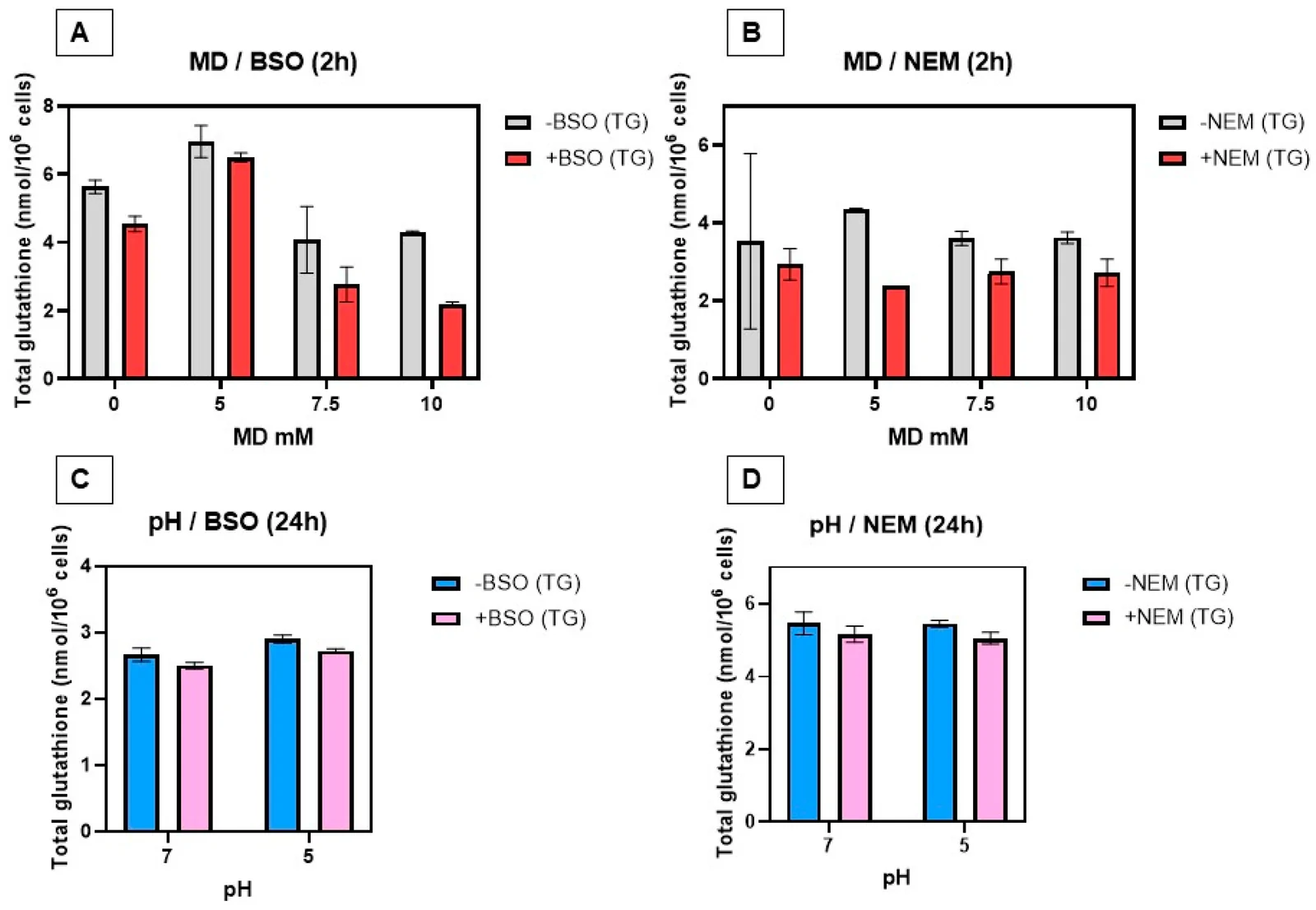
Histograms of total glutathione (TG) levels, determined enzymatically in *T. thermophila* populations treated (2 or 24 h) with MD or pH 5, and in the presence or absence of the inhibitors BSO (**A**,**B**) or NEM (**C**,**D**). Table S5M–P show the statistical analysis of these results.

In the presence of MD, glutathione levels show similar patterns with or without BSO (Figure 9A, Table S5M). An increase in glutathione levels is observed at the lowest MD concentration, followed by a decrease proportional to the MD concentration used. In the NEM treatment, glutathione levels at different MD concentrations do not differ significantly from those observed in the absence of MD (Figure 9B, Table S5N). As with BSO, the presence of NEM reduces average glutathione levels compared to those observed in its absence.

#### 3.2.5. Acid pH Treatment

As shown in Figure 9 panels C,D (Table S5O,P), both BSO and NEM cause a significant decrease in glutathione levels compared to the control. However, in the BSO-treated samples (24 h), there is a significant effect of both BSO and the transition to acidic pH, which significantly increases glutathione levels (Table S5O).

### 3.3. Structural Characteristics and Phylogenetic Insights of T. thermophila GCL, GS and GR Proteins

#### 3.3.1. TthGCL and TthGS Proteins

**Figure 10:**
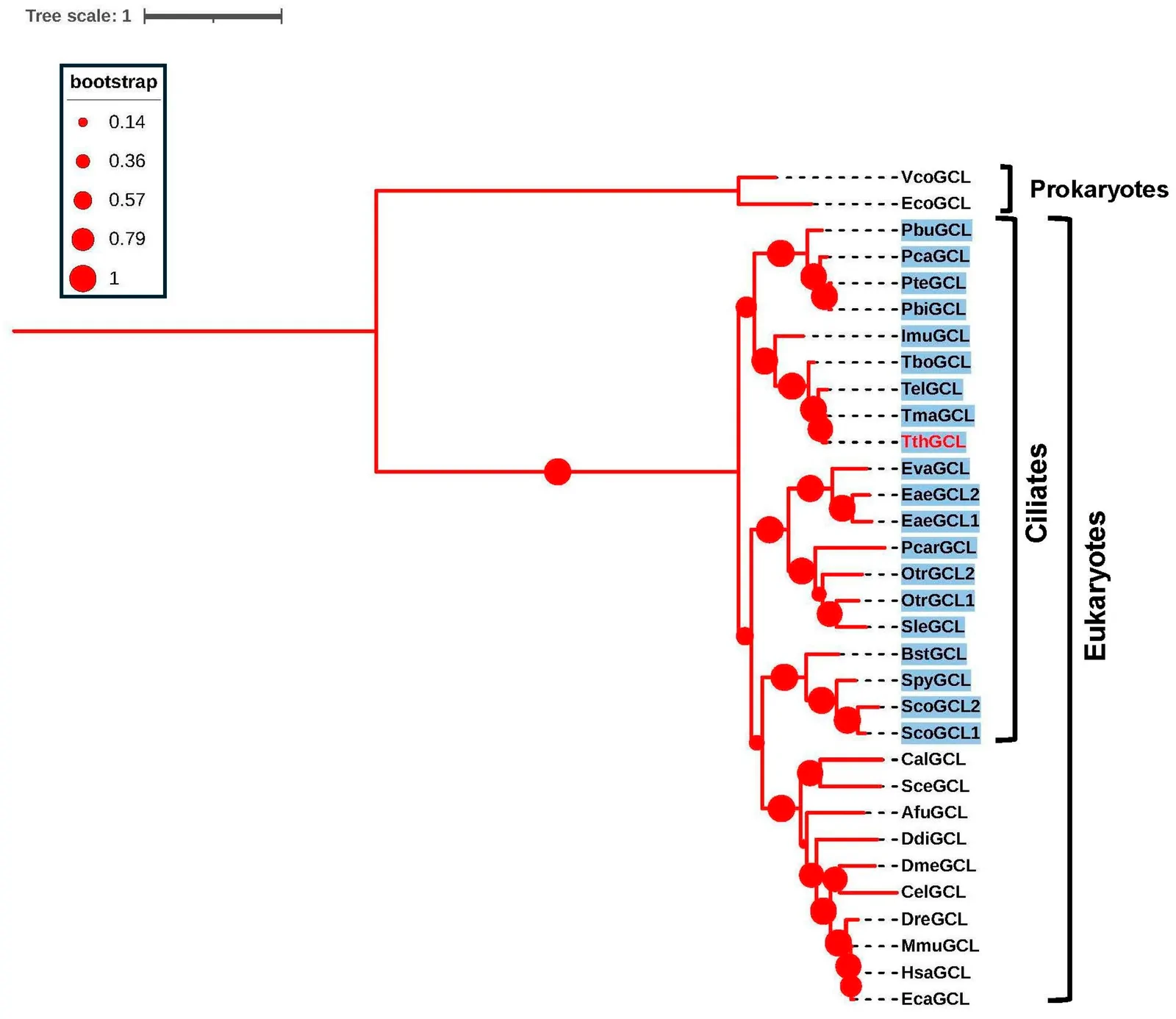
Phylogram of selected eukaryotic and prokaryotic GCL proteins, including TthGCL (highlighted in red). The identification of the eukaryotic GCLs is shown in the Figure S1 caption. Vco (*Vibrio cholerae*); Eco (*Escherichia coli*).

A single gene encoding a GCL (TTHERM_00250970) and another encoding a GS (TTHERM_01049360) were identified in the *T. thermophila* genome, suggesting that both steps of GSH biosynthesis are supported. Among the ciliates analyzed, there are three species (*Oxytricha trifalax*, *Euplotes aediculatus*, and *Stentor coeruleus*) that have two GCL isoforms in their genomes; all the others have only one GCL gene. The protein encoded by the *T. thermophila* GCL gene (which we have named TthGCL) exhibits characteristics typical of many eukaryotic GCLs. TthGCL is 626 amino acids long, which is close to the average size of eukaryotic CGLs (638 aa), and its molecular weight is 73.32 kDa, which is also close to the eukaryotic average (73.66 kDa). Figure S1 shows a partial alignment of the catalytic domain of various GCLs obtained from different ciliate genomes, along with those of other eukaryotic organisms. A high degree of amino acid identity is observed in this region, along with three residues (indicated by red arrows) that potentially interact with cysteine to facilitate its binding to glutamate during the first phase of GSH biosynthesis. This confirms the identification of this protein (TthGCL) as a true GCL enzyme. Figure 10 shows a phylogram of selected eukaryotic and prokaryotic GCLs, including TthGCL. Prokaryotic GCLs differ significantly from eukaryotic GCLs. The GCLs from ciliates are grouped into at least five subgroups based on their taxonomic proximity. Species of the genus *Paramecium* (order Peniculida) constitute one of these subgroups, as do species of *Tetrahymena* and *Ichthyophthirius multifilis* (order Hymenostomatia). Euplotes species (order Spirotrichea) are grouped together and linked to other ciliates of the same order, such as *Oxytricha trifallax*. The last subgroup includes ciliate species of the order Heterotrichida and is linked to the rest of the selected eukaryotes (Figure 10).

**Figure 11:**
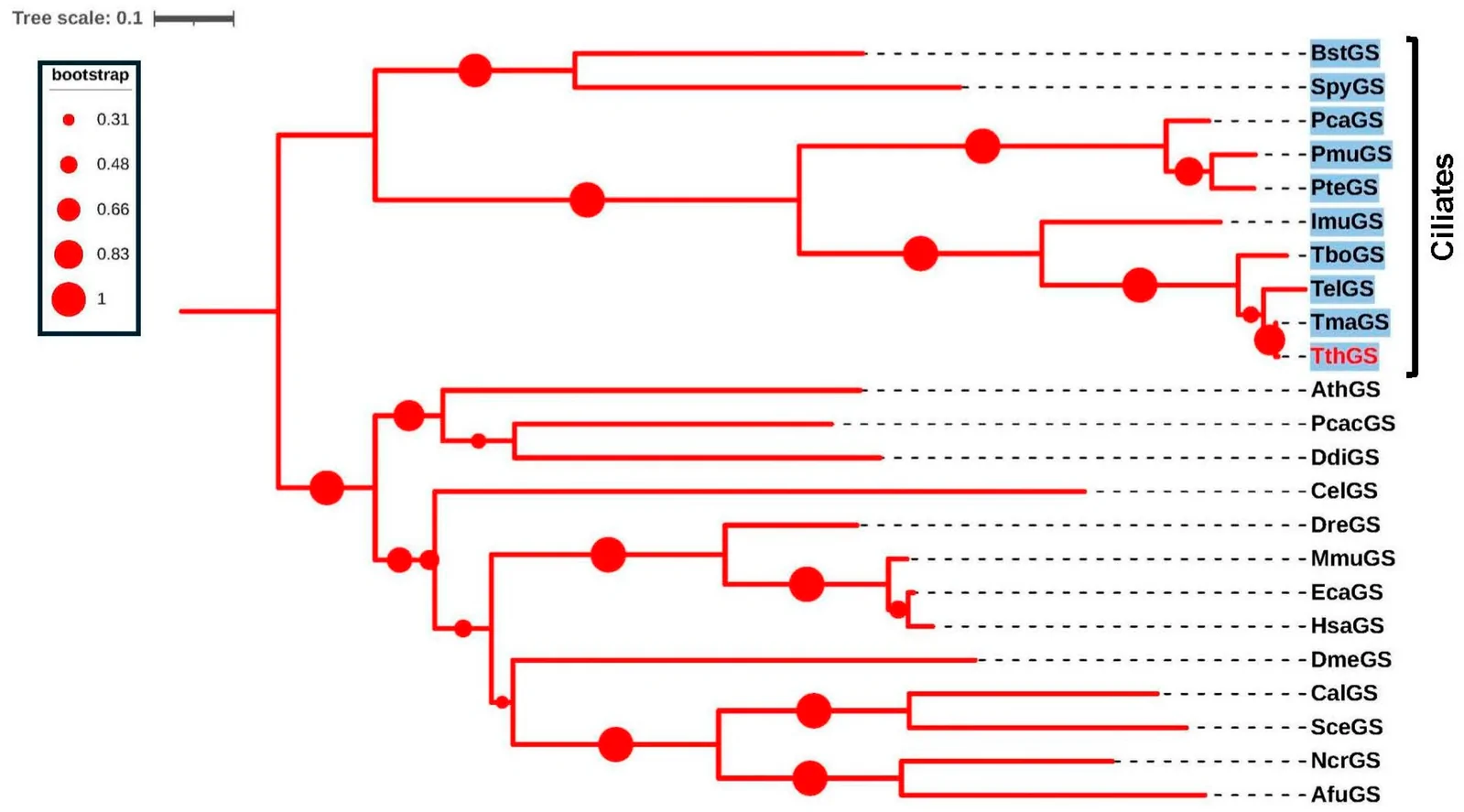
Phylogram from the amino acid sequences of selected eukaryotic GSs, including TthGCL (highlighted in red). The identification of the eukaryotic GSs is shown in the Figure S2 caption.

Partial alignment of the TthGS amino acid sequence with those from other eukaryotes (Figure S2) reveals a high degree of conservation in three loop regions that are important for the activity of these enzymes: the substrate-binding loop (S-loop), the glycine-rich loop (G-loop), and the alanine-rich loop (A-loop) (Figure S2). It confirms that this protein (TthGS) is indeed a glutathione synthase. A single gene encoding this enzyme was detected in all the ciliates analyzed. Figure 11 shows a phylogram from the amino acid sequences of selected eukaryotic GSs. The group of ciliate GS is separate from the GS from other eukaryotes (including fungi, plants, and animals). Likewise, the different species are grouped based on their taxonomic proximity.

#### 3.3.2. TthGR Protein

**Figure 12:**
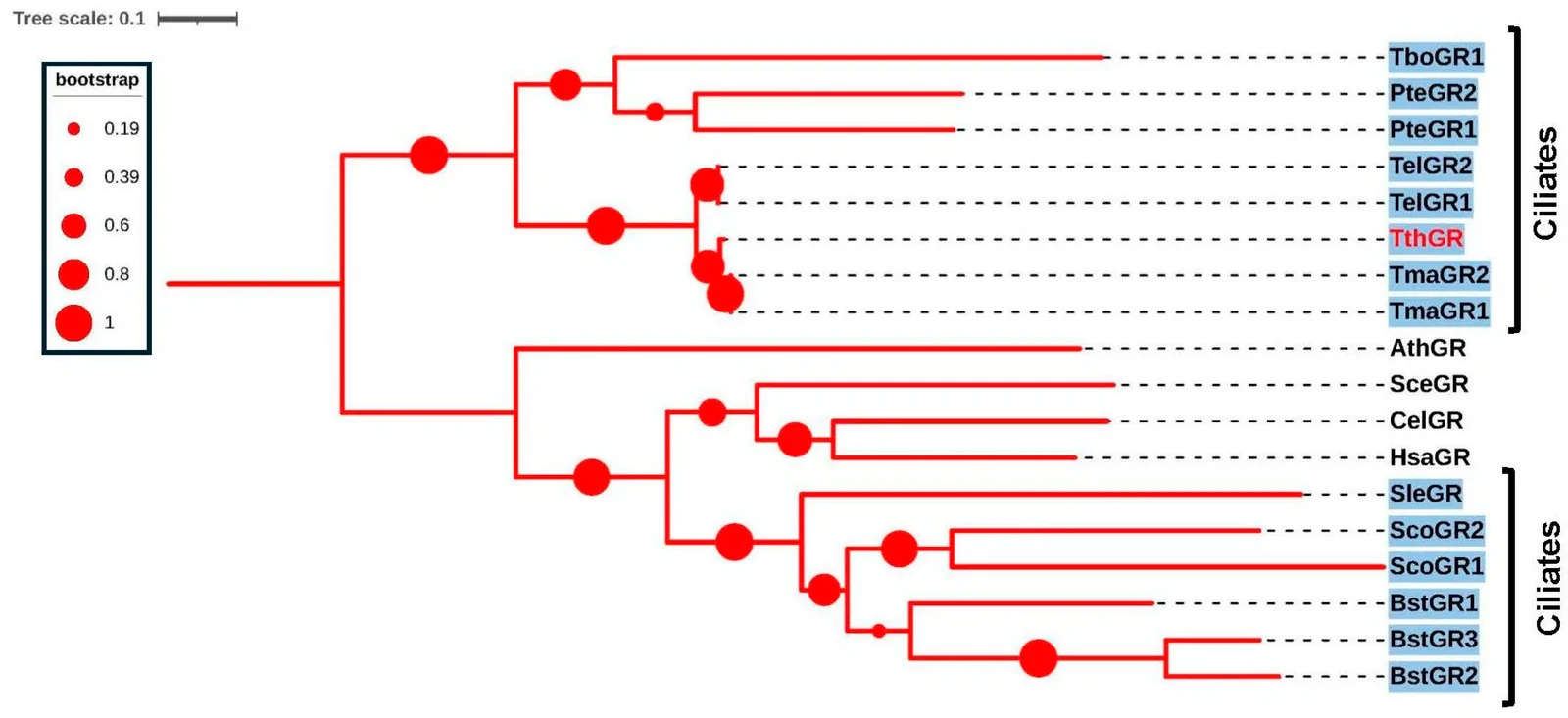
Phylogram illustrating the distribution of selected GR proteins from ciliates, including TthGCL (highlighted in red), and other eukaryotes. The identification of the ciliate GRs is shown in Table S6. Ath (*Arabidopsis thaliana*); Sce (*Saccharomyces cerevisiae*); Cel (*Caenorhabditis elegans*); Hsa (*Homo sapiens*).

Like other organisms, *T. thermophila* has one of the most widespread antioxidant systems (GSH/GR). As with the GCL and GS enzymes, this ciliate has a single gene encoding a GR (which has been named TthGR). The genome of this ciliate contains six genes encoding proteins with domains characteristic of the pyridine–nucleotide–disulfide oxidoreductase family, one of which is TthGR. Table S6 shows the most important structural features of ciliated GRs. Unlike *T. thermophila* and other species, which have only one GR isoform, others have two or even three GR isoforms (Table S6). The average molecular weight of ciliate GRs is 52.4, which falls within the range of GRs. Likewise, TthGR and other ciliate GRs possess the three characteristic domains of PNDR (pyridine–nucleotide–disulfide reductase) enzymes [53]. The highly conserved active site sequence (CXXXXC) (where “X” is any amino acid) among PNDR is equally conserved in ciliate GRs. The FAD-binding domain of ciliate GRs consists of six amino acids with a highly conserved sequence (GXGXXG), as occurs in PNDR enzymes (Table S6). Similarly, the NADPH-binding domain is a highly conserved sequence (XGXXA) in PNDR enzymes and ciliate GRs. Some species have two regions or domains for binding NADPH within the same GR isoform, such as *Tetrahymena elliotti*, *Stentor coeruleus*, and *Blepharisma stoltei* (Table S6). The phylogram shown in Figure 12 illustrates the distribution of selected GR proteins from ciliates and other eukaryotes. Ciliate GRs are divided into two distinct groups based on their taxonomic proximity.

### 3.4. Expression Analysis of TthGCL and TthGR Genes Under Different Abiotic Stressors

**Figure 13:**
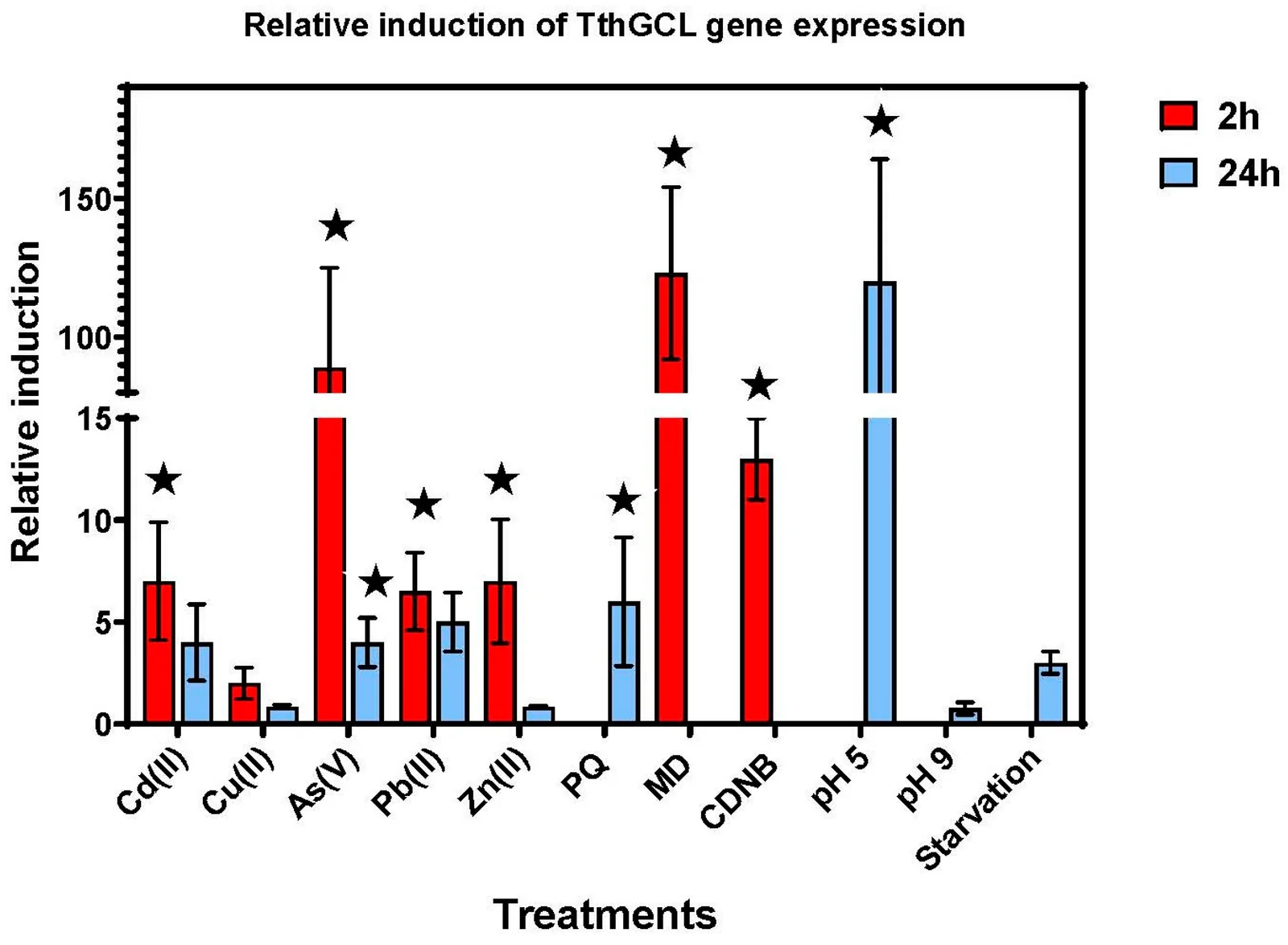
Relative expression levels (qRT-PCR) of the TthGCL gene under different stress treatments (Cd(II), Cu(II), As(V), Pb(II), Zn(II), PQ, MD, CDNB, pH 5, pH 9 or Starvation) (2 or 24 h). Asterisks indicate significant values (*p* < 0.05). The data are shown in Table S7.

We chose TthGCL for analysis by qRT-PCR from among the two genes involved in GSH biosynthesis, as it is the key enzyme in this process. The results of the relative expression levels of the TthGCL gene are shown in Figure 13 and Table S7. Most treatments with metal(loid)s significantly induce the expression of this gene, except for Cu(II). There are notable differences in induction levels depending on the cation and the exposure time. In all cases where induction occurred, short-term exposures (2 h) resulted in higher induction levels than longer-term exposures (24 h) for the same metal(loid) (Figure 13). As(V) is the one that induces the highest induction values (an average of 89-fold of the basal value). Cd(II), Pb(II), and Zn(II) also induce this gene, with similar induction levels after 2 h of treatment (an average increase of 6.6- or 7-fold) (Figure 13, Table S7). In general, prolonged exposures (24 h) significantly reduce induction values compared to those obtained after 2 h, with Pb(II) causing the smallest reduction.

Exposure to oxidizing agents (PQ, MD, or CDNB) also causes overexpression of the TthGCL gene; MD causes an increase of approximately 123-fold compared to basal levels after 2 h treatment. Regarding pH, only acidification (pH 5) significantly induces (by about 120-fold) the expression of the TthGCL gene; in contrast, pH 9 does not induce the expression of this gene. Starvation for 24 h results in a slight (about 3-fold) but non-significant increase in the expression of this gene (Figure 13, Table S7). 

**Figure 14:**
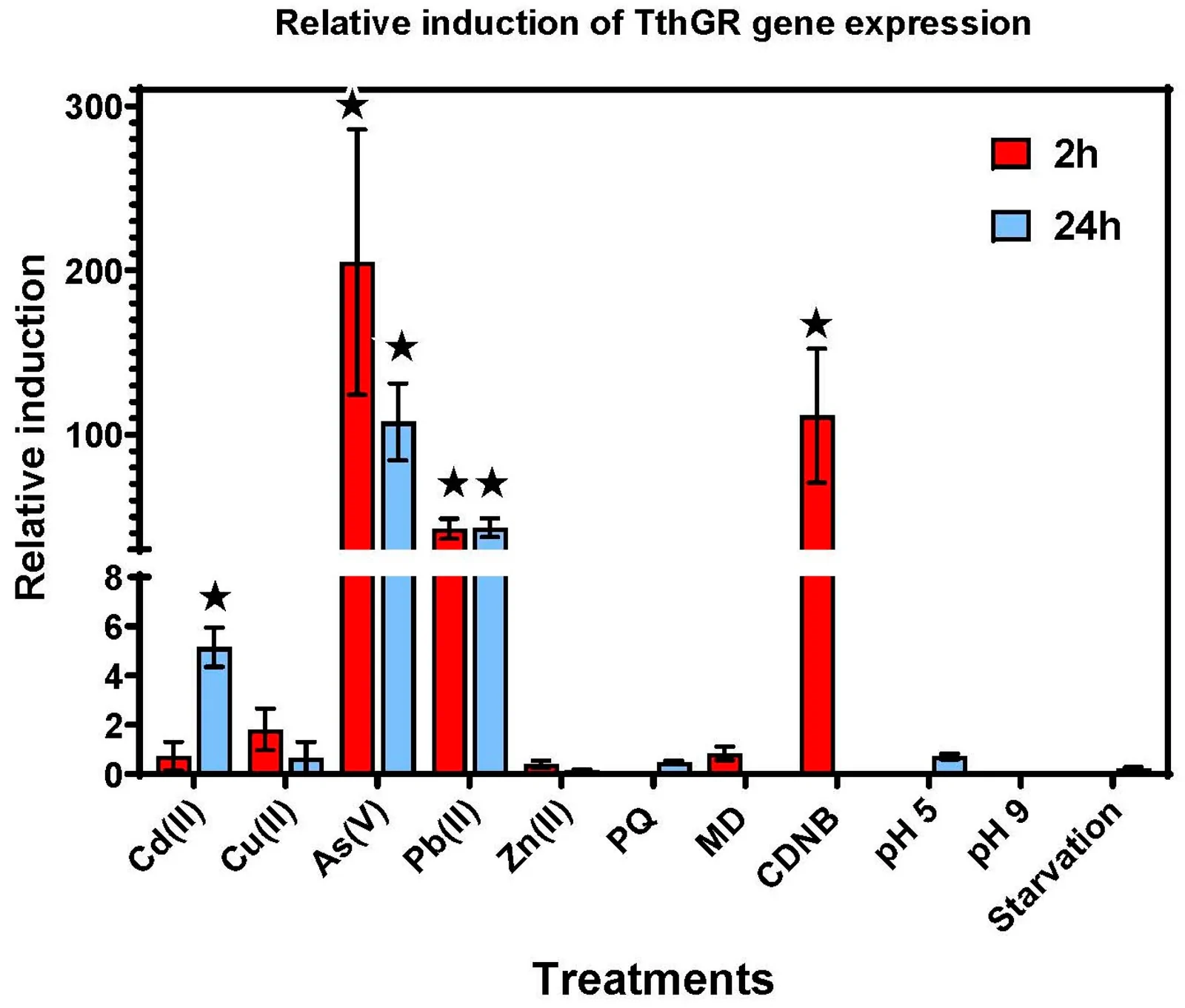
Relative expression levels (qRT-PCR) of the TthGR gene under different stress treatments (Cd(II), Cu(II), As(V), Pb(II), Zn(II), PQ, MD, CDNB, pH 5, pH 9 or Starvation) (2 or 24 h). Asterisks indicate significant values (*p* < 0.05). The data are shown in Table S8.

TthGR is significantly induced by Cd(II) (24 h exposure), and by As(V) and Pb(II) at both 2 and 24 h. It is also strongly induced by CDNB (Figure 14, Table S8).

## 4. Discussion

### 4.1. Biotoxicity of Different Stressors, Effect of GSH or Thiol Group Inhibition, Assessment of Cell Mortality, Thiol Groups and Glutathione

As expected, the toxic effect of each stressor varies depending on the concentration and duration of exposure. In many cases, prolonged treatment (24 h) with a given concentration of a toxic agent causes high mortality, whereas there is virtually no cytotoxicity when exposure lasts only 2 h at the same toxic agent concentrations. Thus, at the concentrations of Cd(II), As(V), or PQ used, no significantly higher mortality rate than that of the control group was detected in the populations treated for 2 h. Similar results under these same conditions were obtained when GSH biosynthesis was inhibited with BSO or thiol groups were blocked with NEM. One exception is the treatment (2 h) with MD, which will be discussed later.

Cadmium is a highly toxic metal to living organisms, even at very low concentrations. One of the main mechanisms involved in the toxicity of Cd(II) is the production of reactive oxygen species (ROS) and, as a result, the induction of oxidative stress [54,55]. As in other living systems, whether unicellular or multicellular, Cd(II) generates various types of ROS in *T. thermophila*, such as superoxide and peroxides [56,57]. The results on the cytotoxicity of Cd(II) in *T. thermophila* confirm our previous findings [56], in which toxicity levels initially remain relatively low (<20% population mortality) until, starting at a certain concentration, a drastic increase in mortality occurs (>90% population mortality). Initially, cellular levels of detoxification mechanisms are sufficient to maintain the viability of a large portion of the population, but once a certain concentration of Cd(II) is reached, this threshold is exceeded, leading to a rapid decline in cell viability.

Inhibition of GSH by BSO leads to a decrease in cell death when cells are exposed to a higher concentration of Cd(II) (1.76 μM). Therefore, a decrease in intracellular GSH levels appears to increase resistance to Cd(II). However, this dramatic decrease in cell death does not occur when cells treated with the same concentration of Cd(II) are exposed to the −SH group blocking agent (NEM). Furthermore, exposure to NEM causes an increase in cell death in the presence of a lower concentration of Cd(II) (1.54 μM). Under these conditions, cell mortality is more than five-fold higher than in the control group (exposed to the same Cd(II) concentration but without NEM).

Contradictory results have been reported regarding the role of MTs under conditions of GSH deficiency or limitation and under the presence of Cd(II) or other oxidizing agents. Some authors, using animal cells or yeasts, have observed that treatment with BSO increases cellular sensitivity to metals and other oxidizing agents [58,59,60]. However, in other studies with animal cells, the removal or limitation of GSH leads to greater resistance to the lethal effects of Cd(II) or to oxidative stress generated by other toxic agents. This phenomenon, observed in both vertebrate and invertebrate animals, appears to be due to an increase in specific metal-binding proteins (CdMTs) that act as antioxidants and interact with the GSH/GSSG system [61,62,63,64]. In addition, knockout mice lacking MT-encoding genes are highly sensitive to oxidative stress [65]. A knockout strain of *T. thermophila* in the MTT1 gene (which encodes a CdMT) [66] has an LC_50_ value that is half that of the control strain, indicating a significantly higher sensitivity to this metal.

From these results, we can infer that GSH is an important antioxidant in the detoxification of Cd(II) in the ciliate *T. thermophila*. GSH appears to be effective when cells are exposed to low concentrations of Cd(II) during both short-term and longer-term treatments. However, at higher concentrations of Cd(II) (for 24 h), and when GSH biosynthesis is inhibited by BSO, CdMTs would play an important role in the response to Cd(II).

When using NEM, higher fluorescence is always detected in the control, regardless of the concentration and exposure time of Cd(II). In general, the presence of NEM leads to increased cell death at various concentrations of Cd(II).

Taken together, the results appear to indicate that *T. thermophila* exhibits an early response to Cd(II) through the involvement of GSH, by directly chelating Cd(II) and/or by reducing the oxidative stress caused by this metal. Furthermore, the CdMTs from this ciliate [67,68] act simultaneously with GSH, reinforcing the cellular response to Cd(II). qRT-PCR analysis has shown that the expression of most *T. thermophila* CdMT genes is strongly and preferentially induced by Cd(II) [69]. The biosynthesis of these CdMTs could explain the notable increase, compared to the control, in thiol groups (CMFDA fluorescence) detected when cells are exposed to Cd(II).

In *T. thermophila*, arsenate As(V) is more toxic than arsenite As(III) [70]. Results show that the mortality of *T. thermophila* populations at each concentration of As(V) is similar whether inhibitors are present or absent. This occurs regardless of the type of inhibitor (BSO or NEM) or the treatment period. However, in all cases, exposure to As(V) for 24 h resulted in higher mortality than exposure for 2 h. This could mean that GSH and other molecules containing thiol groups do not play a significant role in the ciliate response to As(V) stress. This statement contradicts the gene expression data for enzymes involved in GSH biosynthesis and metabolism, such as GCL, GR (which are analyzed in this study), and certain GSTs [13], as well as *Tetrahymena* MTs (MTT1 and MTT5), whose transcription is significantly induced by As(V) [70].

Due to arsenic’s high toxicity, *Tetrahymena*, like other organisms, appears to have developed resistance mechanisms against various chemical forms of arsenic. The biotransformation of arsenic is one of the most extensively studied detoxification mechanisms [71]. Two main mechanisms have been described: As(III) or As(V) enzymatic methylation [72], and reduction of arsenate to arsenite, which is then ejected from the cell by an arsenite-specific efflux pump [73]. *T. thermophila* can reduce arsenate to arsenite and excrete it from the cell [74]. Likewise, two species of *Tetrahymena* (*T. thermophila* and *T. pyriformis*) have the ability to biomethylate both As(V) and As(III) [74,75,76]. All of these mechanisms could contribute to the cellular response to As(V), along with GSH and MTs.

With regard to PQ, the generation of ROS is considered to be the primary cause of its biotoxicity [77]. Given that PQ generates various types of ROS in both animals and plants, GSH is thought to play a key role in the stress response caused by this herbicide [78,79]. However, it has been shown that *T. thermophila* exhibits unusually high resistance to PQ [39], and GSH does not appear to play a significant role in defense against this toxic agent. Thus, the use of BSO does not result in any difference in mortality compared to populations without BSO during exposure (2 h) to PQ. However, in 24 h treatments, there is a slower increase in mortality in the presence of BSO, although only at certain concentrations (2.5 and 5 mM). With BSO, a greater decrease in thiol group content is observed 2 h after exposure to PQ, but levels remain similar as those in the controls at 24 h (PQ + BSO).

In mammals, the presence of BSO causes a dramatic increase in the cytotoxicity of PQ [80]. The limited role of GSH as an NEM antioxidant in this ciliate is once again evident in the results obtained in the presence of NEM. The presence of this −SH group blocking agent does not significantly increase PQ-induced mortality, in either 2 or 24 h treatments. Furthermore, it reduces the −SH groups compared to PQ treatments without NEM.

The MT genes from *T. thermophila* do not appear to be induced by PQ, with the exception of the MTT5 gene, which is induced about 6-fold above the basal level [38]. Induction of MT genes by PQ has been detected in various animal cell types, including humans [81,82,83,84]. The trigger for MT gene induction appears to be mitochondrial dysfunction, one of PQ’s target organelles [85,86]. This MT induction appears to be greater when GSH biosynthesis is blocked with BSO [87,88,89].

The MD toxicity resides mainly in the generation of superoxide anions and hydrogen peroxide, although the exact mechanism of its biotoxicity remains unknown [90,91]. Furthermore, studies in animal cells have shown that MD is mutagenic and carcinogenic [92]. Although studies are scarce and comparisons are difficult to make due to differing experimental conditions, *Tetrahymena* appears to be more resistant to MD than some animal cells [93], and even than certain anaerobic protists such as *Hexamita* and *Giardia intestinalis* [94,95]. The results show that MD always reduces GSH levels compared to the control, regardless of the presence or absence of BSO or NEM. Nevertheless, both GSH levels and −SH groups, in the presence of high MD concentrations, are consistently lower in populations exposed to BSO or NEM than in those not exposed to these inhibitors.

From these results, we can infer that MD causes GSH consumption in proportion to the concentration of the oxidizing agent used. GSH consumption in the presence of MD appears to be a quite widespread phenomenon among organisms. Various studies carried out with bacteria, filamentous fungi, yeasts, animals, and plants have detected a decrease in free GSH as a result of exposure to MD [96,97,98,99,100]. In fact, in some studies, MD has been intentionally used to reduce GSH [101]. Two possible, non-mutually excluding mechanisms have been proposed to explain the decrease in GSH caused by MD: (1) the direct interaction of GSH with free radicals generated by exposure to MD [102], and (2) the direct conjugation of GSH with MD in a process mediated by specific GST enzymes. These conjugates have been detected in the yeast *Saccharomyces cerevisiae* [103]; they are incorporated into vacuoles and eventually expelled from the cell by ATP-dependent transport pumps. The formation of MD-GSH conjugates appears to be the main mechanism of MD detoxification in yeasts, leading to the induction of GST gene expression [104]. In *T. thermophila*, MD (2 h treatment) induces at least four cytosolic GST genes, all of which are also induced by other stressors. Of these, the one showing the highest induction level is TthGSTM27 (one of the 54 Mu-class GSTs) [13].

Acidification of the culture (for 24 h) does not result in significant differences in cell mortality, whether in populations exposed to BSO or NEM or in those not exposed to these inhibitors. Regarding thiol group content, only a slight significant increase is observed in populations exposed to an acidic pH (without BSO) and a decrease in the presence of BSO. NEM and acidic pH decrease thiol groups, while acidic pH (without NEM) remains the same as the control. GSH levels remain similar (with or without BSO or NEM) compared to the controls.

There are very few studies on the effect of pH on cellular GSH levels, and the few that have been published appear to be contradictory. In the yeast *Candida utilis*, acid stress increases intracellular GSH levels, but this increase is only temporary because, under these conditions, the rate of GSH efflux also increases [105,106]. In the bacterium *Rhizobium tropici*, an increase in GSH synthesis has also been observed in response to a pH decrease [107]. In animal cells, a decrease in GSH levels is observed following acidification of the medium [108,109].

### 4.2. Assessment of Total Cellular Glutathione (GSH/GSSG) Throughout the Growth Curve and Under Stress Conditions (with or Without BSO or NEM Inhibitors)

Throughout the *T. thermophila* growth curve, total glutathione levels remain constant during the latency and accelerated growth phases. During the exponential phase (lasting approximately 12 h), glutathione levels increase about three-fold, reaching a peak at the end of this phase. During the stationary phase, glutathione levels decrease by about two-fold.

Miao et al. [110] have developed a microarray of the macronuclear genome from *T. thermophila* and analyzed the expression of various genes (including TthGCL) under different conditions (growth–division cycle, conjugation, and starvation). According to this study, GSH levels increase significantly during the exponential phase and decrease during the stationary phase; these results are consistent with those reported in the present study. In animal cell cultures, GSH levels increase prior to entry into the S phase (DNA synthesis) [111]. GSH provides the reducing power necessary for ribonucleotide reductase activity via glutaredoxins or thioredoxins, and this is a rate-limiting step in DNA synthesis [112]. Therefore, the increase in GSH during the exponential phase of a cell population is a result of increased DNA synthesis, which is necessary for cell proliferation. The decrease in GSH levels during the stationary phase may be due to the absence of or reduction in DNA synthesis, some cell death, and a deficiency of nutrients (primarily methionine, from which cysteine is derived), which inhibit cell proliferation and GSH biosynthesis. Furthermore, during this phase, cells undergo oxidative processes (aging), consuming pre-existing GSH [113].

Table 1 summarizes the overall results obtained from flow cytometric and enzymatic analyses, showing whether or not there was an interaction between the stressor and the inhibitor used (BSO or NEM), as well as the effect on the three parameters analyzed (cell death, −SH groups, and total glutathione).

Cd(II)-induced stress increases intracellular levels of molecules containing −SH groups, as well as total intracellular glutathione (GSH/GSSG) levels. Similarly, the presence of As(V) appears to increase both molecules containing −SH groups and glutathione during short exposure times (2 h), whereas during prolonged exposure (24 h), both parameters decrease. This decrease can be explained by the following facts: As(V) can be reduced to As(III), and the reducing power for this process is derived from GSH, which is converted to its oxidized form (GSSG) [114]. Several GSH molecules can chelate an As(V) atom [115], thereby decreasing the amount of free GSH. This can also act as a cofactor for various enzymes involved in redox reactions to reduce the ROS formation caused by arsenic [116].

The expression of MT genes under arsenic stress, involving As(III) or As(V), has been studied almost exclusively in human and mouse cells [117]. In all cases analyzed, an increase in the expression of certain MT genes has been detected. The possible interaction between the −SH groups from MTs and arsenic cations has only been studied using As(III). In a recombinant MT human (MT1A), it has been shown that As(III) binds to the −SH groups of the cysteine residues of this MT with the stoichiometry As(III)_6_MTCys_20_ [117]. Likewise, MTs can bind both monomethylated and dimethylated forms of As(III) [118]. Therefore, the formation of stable MT-As(III) complexes could be an additional detoxification mechanism.

With regard to As(V), its potential interaction with MTs has not been studied, although we have indirect evidence that it most likely exists as well. The MT genes encoding the MTT1 and MTT5 from *T. thermophila* show increased expression primarily following 24 h treatment with As(V) [70]. The importance of MTs’ role in counteracting the toxic effects of As(V) in *T. thermophila* is corroborated by the GFPMTT1 and GFPMTT5 strains of this ciliate, which have a higher number of copies of each MT gene (MTT1 and MTT5, respectively) and exhibit greater resistance to both As(III) and As(V) (their LC_50_ values are approximately 16- and 3-fold higher, respectively, than that presented by the wild-type strain) [70]. Similarly, the MTT1KO (a strain lacking the MTT1 gene) and MTT5KD strain (with a reduced number of copies of the MTT5 gene) exhibit significantly lower LC_50_ values than the wild-type strain, indicating that these genetic alterations make these strains more sensitive to both As(III) and As (V) [70].

Due to the high toxicity of As(V) in *T. thermophila*, during prolonged exposure both GSH and MTs (primarily MTT5) would be involved, which would corroborate the results of the enzymatic assays obtained.

In general, PQ appears to decrease −SH groups, while glutathione either decreases or maintains levels comparable to those in the control (Table 1). Several studies have reported that PQ decreases cellular GSH levels mainly due to NADPH oxidation, such that the GR enzyme cannot reduce GSSG [78,80,115,119]. PQ induces (about 61-fold) the expression of the TthGSTM32 gene among a total of five GST genes that are also expressed in response to this herbicide [13], which could imply the transfer of GSH to PQ, thereby inactivating it, and resulting in a decrease in cellular GSH levels.

Nakagawa et al. [120], using mouse liver cells, found that treatment with PQ following inhibition of GSH biosynthesis with BSO led to an increase in MT synthesis, which was not observed after treatment with PQ alone. However, in *T. thermophila*, the MTT5 gene is induced about 9-fold in the presence of PQ (24 h treatment) [38].

MD, which primarily induces oxidative stress, reduces both parameters (total glutathione and −SH groups) (Table 1). MD significantly induces the expression of at least four TthGST genes of three types (TthGSTM26, TthGSTM27, TthGSTO7, and TthGSTN1); therefore, some of them, such as TthGSTM26 (which exhibits the highest expression level, 5-fold) [13] or several of them, could be involved in the formation of MD-GSH conjugates.

An acidic pH (pH 5) does not substantially alter the levels of molecules containing −SH groups or total glutathione, although a certain effect (decrease) is observed with BSO regarding −SH groups (Table 1).

From Table 1, which provides an overview of the results regarding stressors and inhibitors in relation to cell mortality, −SH groups, and total glutathione, we can infer the following general points: 1—The highest number (81%) of interactions between inhibitors and stressors with respect to any analyzed parameter corresponds to the −SH groups. This is followed by 37% for total glutathione, and by cell death at only 25%. 2—Most of the stressors used increase cell death compared to the control, regardless of whether or not an inhibitor (BSO or NEM) is present, except in the case of acidic pH. 3—Regardless of the stressor, the presence of the inhibitor (BSO or NEM) reduces the levels of intracellular −SH groups in most cases (about 73% of cases). 4—Regardless of the stressor, the presence of BSO increases the intracellular total glutathione content in most cases. In contrast, NEM both increases and decreases this parameter in a similar number of cases, depending on the stressor type and the time of exposure.

### 4.3. TthGCL, TthGS and TthGR: A Comparative Analysis

#### 4.3.1. TthGCL and TthGS Enzymes

In eukaryotic organisms, GSH biosynthesis involves two steps (both ATP-dependent), catalyzed by the enzymes GCL (first step) and GS (second step). However, the first of these enzymes (GCL) plays a crucial role [121], and there are several reasons to support this: 1—Some Gram-positive bacteria, such as *Streptococcus agalactiae*, possess a bifunctional GCL that catalyzes both phases. A similar GCL gene is also present in certain species from the genera Listeria, *Clostridium*, *Enterococcus*, and *Pasteurella* [122,123]. 2—In both plants and bacteria, the dipeptide (glutamyl-cysteine) can replace the tripeptide (glutamyl-cysteinyl-glycine, or GSH) under environmental stress conditions [31,124]. However, both GCL gene knockout strains and those with low levels of this enzyme (due to the use of RNA interference) or cells exposed to GCL inhibitors (such as BSO) are highly sensitive to toxins or environmental stress conditions [30,125,126,127]. This hypersensitivity to toxic stress is abolished by adding GSH or glutamyl-cysteine to the medium. 3—The reaction catalyzed by GCL is the rate-limiting step in GSH biosynthesis and therefore determines the amount of GSH synthesized. In mammals, this enzymatic activity is highly regulated. There are three levels of regulation: transcriptional (in response to multiple agents causing oxidative stress), post-transcriptional (mRNA stability, GCL phosphorylation, and/or caspase-dependent GCL cleavage during apoptosis) [29,30], and at the metabolic level, both through competitive (non-allosteric) feedback inhibition by the end product (GSH), which involves the binding of GSH to GCL, and through the availability of the precursor L-cysteine [30]. 4—Both GSH and the dipeptide (γ-glutamyl-cysteine) are precursors of phytochelatins (oligopeptides involved in metal chelation) [22,128]. Transgenic plants and yeasts overexpressing the GCL gene are more resistant to certain toxic metals (Cd, As or Hg) [129,130,131]. These four reasons confirm the great importance of this enzyme (GCL) in the GSH metabolism, a tripeptide found in most living organisms.

The *T. thermophila* genome contains a single gene encoding GCL, as is the case with other eukaryotes, such as *Trypanosoma brucei* [132] or *Schizosaccharomyces pombe* [133]. As shown in the alignment of Figure S1 (which includes 17 ciliates, among other eukaryotes), GCLs show a clear catalytic domain. The partial amino acid sequences (Figure S1) indicate the three amino acid residues that interact with cysteine to catalyze GSH.

We have not found a gene encoding a modifier or regulatory GCL subunit in the *T. thermophila* genome, unlike in some mammals and insects, where GCL is heteromeric (one catalytic subunit and one regulatory subunit), with the latter having a regulatory function in catalytic activity by reducing feedback inhibition by the final product (GSH) [14,30]. Therefore, we can conclude that the *T. thermophila* GCL is monomeric, most likely as in other ciliates. In the parasitic protozoan *Plasmodium berghei*, a GCL has been characterized as heterodimeric [134].

According to Copley and Dhillon [135], two possible scenarios could be considered regarding the evolution of GCLs: 1—The GCL gene could have originated early in both the prokaryotic and eukaryotic lineages and undergone horizontal transfer from one domain to the other, and 2—the GCL gene could have been present in the last universal common ancestor (LUCA) and been lost early in some archaea, at the origin of the eukaryotic lineage, and in many bacteria. This second possibility requires that the GCL gene originated in LUCA before the appearance of oxygen, which occurred after the evolution of cyanobacteria (~2600 million years ago). On the contrary, the possibility that the GCL gene arose early in the bacterial lineage and was later transferred to the eukaryotic lineage and to certain archaea (such as *Halobacterium*) seems more plausible [135].

Phylogenetic analysis of selected GCL sequences (Figure 4) reveals a clear separation between prokaryotes and eukaryotes; among eukaryotes, ciliates are positioned closest to the prokaryotes and are separated from the rest of the eukaryotes. Doolittle [136] proposed a mechanism for lateral gene transfer between domains based on the idea that protists that feed on bacteria can incorporate bacterial genes into their eukaryotic genomes. Since most ciliates can feed on bacteria, this possibility could hold true.

Some ciliate species (*Euplotes aediculatus*, *Oxytricha trifallax*, and *Stentor coeruleus*) possess two very similar GCL paralogous genes, unlike the rest, which have only one copy, a situation that is uncommon in ciliate genomes composed of gene families.

*T. thermophila*, like other ciliates, also possesses the second enzyme (GS) involved in the second reaction of GSH catalysis. In all of the ciliates analyzed, it exists as a single gene. This enzyme (TthGS) possesses the three conserved loop regions found in many other eukaryotic GS enzymes. Furthermore, the resulting phylogenetic tree completely separates the ciliate sequences from those of other highly diverse eukaryotes.

#### 4.3.2. TthGR Enzyme

As in most aerobic organisms, *T. thermophila* and other ciliate species have the GSH/GR antioxidant system. The GR enzyme catalyzes the conversion of GSSG to 2GSH in the presence of NADPH [137]. This enzyme plays a crucial role in maintaining the GSSG/2GSH ratio, thereby controlling cell cycle regulation and other processes (such as apoptosis) [9].

As with the GCL and GS enzymes, *T. thermophila* possesses a single copy of the TthGR gene, unlike some ciliates (including other *Tetrahymena* species), which have two or three paralogous genes encoding this enzyme (see Table S6). The TthGR protein exhibits the three characteristic structural domains of the PNDR enzyme family, confirming its identification as a GR.

Phylogenetic analysis of selected GR sequences from ciliates and other eukaryotes reveals a clear division into two groups: one group comprising members of the class Oligohymenophorea (*Tetrahymena* and *Paramecium*) and another comprising the classes Heterotrichea (*Stentor* and *Blepharisma*) and Spirotrichea (*Stylonychia*), which share a common origin with other eukaryotes. Interestingly, the GR genes in the first group (Oligohymenophorea) contain introns (2–5), whereas those in the other two classes of ciliates do not contain introns (see Table S6).

### 4.4. TthGCL and TthGR Gene Expression Under Different Stress Conditions

The qRT-PCR results show that the TthGCL gene responds significantly and early (within 2 h) to agents that induce oxidative stress. The induction ranking is MD > As(V) > CDNB > Cd(II) > Zn(II) > Pb(II), after 2 h of treatment. However, after 24 h of treatment, the ranking of significant induction values changes considerably: pH 5 >> PQ > As(V).

This is consistent with previous studies on GCL in other organisms, in which oxidative stress caused by various agents is associated with increased levels of GSH, GCL activity, and transcription levels [32,138]. MD is a compound that induces the formation of ROS, such as superoxide, peroxide, and hydroxyl radicals [139]. Likewise, MD has previously been described as an inducer of GCL gene expression [29,140]. Among the metal(loid)s tested, the arsenate cation As(V) is the one that most significantly induces the expression of the TthGCL gene. Both the maintenance of glutathione levels with or without BSO, and their increase in the absence or presence of NEM, could be explained by the increased expression of the TthGCL gene at 2 or 24 h after treatment with As(V). In addition to this early increase in the TthGCL gene, there is also an increase in the expression of MT genes such as MTT1 and MTT5, particularly at 24 h [70], which could compensate for smaller but still significant TthGCL gene expression.

Other metals (Cd, Zn, or Pb) also significantly induce the TthGCL gene during a 2 h treatment; in the case of Cd(II), this would maintain glutathione values at control levels, with or without BSO. However, as in the case of As(V), exposure to these metals (Cd, Zn, or Pb) involves the intervention of MTs, particularly MTT5 and MTT1, in both short (2 h) and long (24 h) exposures [67,141].

The xenobiotic CDNB induces (ranked 3rd) TthGCL gene expression and is also a compound that causes oxidative stress. The herbicide PQ, another oxidizing agent that generates superoxide anions that form H_2_O_2_ and hydroxyl radicals, also significantly upregulate the expression of the TthGCL gene, but only 24 h after exposure. This is consistent with the total glutathione levels, which remained the same as the control both in the absence and in the presence of BSO or NEM. In yeast, a similar effect of PQ on the GCL gene expression has been reported [142]. Another herbicide, similar to PQ, called Diquat (DQ), also induces the expression of GCL genes [143]. H_2_O_2_ also induces the expression of this gene [29]. In general, oxidants deplete GSH by lowering its intracellular levels; therefore, to maintain optimal GSH levels, its biosynthesis—and thus the primary enzyme (GCL) involved in this process—must be induced.

pH 5 increases the expression of the TthGCL gene, with an induction value similar to that of MD, but after 24 h of treatment. This would be consistent with GSH levels remaining similar to those of the control, with or without BSO or NEM treatments. This is the first time it has been shown that a eukaryotic microorganism, such as *T. thermophila*, significantly induces the expression of a GCL gene in response to acidic pH. In contrast, basic pH (pH 9) does not significantly induce the expression of this TthGCL gene, nor does starvation (24 h treatment). Very little is known about the role of GSH in acid stress; however, it has been reported that in bacteria, GSH protects the cell against acid stress [144,145]. The optimal pH for GCL is slightly basic (pH 8.2) in *Drosophila* [146]. As pH decreases, enzyme efficiency decreases, such that the same amount of enzyme generates less GSH, which would trigger induction of GCL gene expression to maintain optimal intracellular GSH levels.

The TthGR gene is significantly upregulated (at 2 and 24 h) under As(V) stress, which coincides with the findings for the TthGCL gene. Arsenic is a major enzymatic inhibitor of the mitochondrial respiratory process capable of generating ROS and RNS (reactive nitrogen species), but primarily hydrogen peroxide, superoxide, singlet oxygen, peroxyl radicals, nitric oxide, and dimethyl arsenic peroxide radicals [147]. Therefore, GSH would play an important role in counteracting the toxic action of this metalloid. Enzymes involved in its biosynthesis and regeneration (return to the reduced state) are intensified, counteracting the high oxidative toxicity of arsenic.

The ranking of the most significant induction values for TthGR gene expression at 2 h post-treatment is As(V) > CDNB >> Pb(II), while at 24 h it is As(V) >> Pb(II) > Cd(II). Both As(V) and Pb(II) appear in the rankings of induction values for the TthGR gene. Furthermore, As(V) and CDNB appear in the rankings (after 2 h treatment) for both the TthGCL and TthGR enzymes. As(V) could inhibit both enzymes, hence the need to overexpress their genes and produce greater enzyme amounts, in addition to its multiple oxidative effects.

Studies using transgenic plants have shown that GR plays a prominent role in cellular resistance to oxidative stress caused by ozone, metals, salinity, thermal shock, etc. GR activity increases in various plants following exposure to Cd(II) [148].

A comparative analysis of the fold-induction levels from both genes (TthGCL and TthGR) in response to the same stressor is shown in Table 2. This comparison suggests that a greater amount of TthGR is required for stressors that cause significant oxidative stress (such as As(V), Pb(II), or CDNB) by oxidizing glutathione. By contrast, other stressors require the biosynthesis of more GSH, increasing the amount of TthGCL, such as MD or acidic pH.

## 5. Conclusions

From the results of this study, the following general conclusions can be drawn: 1—Like other eukaryotes, *T. thermophila* has in its genome the two main enzymes (GCL and GS) involved in GSH biosynthesis. It also has one GR that restores the reduced state of GSSG molecules. Bioinformatic analysis reveals that this is also common among other ciliates. We have not found a gene encoding a modifier or regulatory GCL subunit in the *T. thermophila* genome. Therefore, it can be inferred that the enzyme is monomeric. 2—Since −SH groups are found not only in GSH but also in other molecules involved in the cellular response to oxidative stress, the greatest number of interactions between stressors and inhibitors occurs when this parameter is measured. 3—Almost all the stressors used increased cell mortality compared to the control (without the stressor), regardless of treatment with BSO or NEM, except for acidic pH. 4—Regardless of the stressor, the presence of the inhibitor (BSO or NEM) reduces intracellular levels of −SH groups in the great majority of cases. This is consistent with the expected action of these inhibitors. In many cases, the presence of the GSH biosynthesis inhibitor (BSO) increases the amount of total intracellular glutathione, which is explained by the induction of GCL gene expression under most of the stressors used. In contrast, the −SH group inhibitor (NEM) can either increase or decrease the amount of total glutathione, depending on the stress treatment. 5—Oxidative stress caused by certain stressors (As(V), Pb(II), or CDNB) requires the induction of the TthGR gene to reduce GSSG, while others (Cd(II), Zn(II), PQ, or MD), which are less able to induce oxidative stress, preferentially induce the TthGCL gene to biosynthesize more GSH. 6—Phylogenetic analysis results have shown that the three ciliate enzymes analyzed (GCL, GS, and GR) are grouped according to their taxonomic proximity. 7—Taken together, all the results obtained from the ciliate *T. thermophila* have shown the importance of GSH, along with other molecules containing −SH groups (such as metallothioneins), in cellular defense against oxidative stress caused by metal(loid)s and other chemical stressors. It is highly likely that this conclusion can be extended to other ciliates.

## Figures and Tables

**Table 1:** Summary of results obtained from flow cytometric and enzymatic analyses, showing whether there was an interaction between the stressor and the inhibitor used (BSO or NEM), and the effect on the three parameters analyzed (cell death, −SH groups, and total glutathione).

Treatment	Mortality		Thiol Groups		Total Glutathione	
	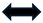	Effect	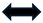	Effect	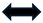	Effect
Cd(II)−BSO (2 h)	No	−BSO (=) +BSO (▲)	Yes	−BSO (▲) +BSO (▲) ^1^	No	−BSO (=) +BSO (=)
Cd(II)−BSO (2 4h)	Yes	−BSO (▲) +BSO (▲)	Yes	−BSO (▲) +BSO (▲)	Yes	−BSO (▲) +BSO (▲)
Cd(II)−NEM (2 h)	No	−NEM (▲) +NEM (▲)	No	−NEM (=) +NEM (▼)	Yes	−NEM (▲) +NEM (▼)
Cd(II)−NEM (24 h)	Yes	−NEM (▲) +NEM (▲)	Yes	−NEM (▲) +NEM (▲)	No	−NEM (▲) +NEM (▲)
As(V)−BSO (2 h)	No	−BSO (▲) +BSO (▲)	Yes	−BSO (▲) +BSO (▲)	Yes	−BSO (▲) +BSO (▼)
As(V)−BSO (24 h)	No	−BSO (▲) +BSO (▲)	Yes	−BSO (▼) +BSO (▼)	No	−BSO (=) +BSO (=)
As(V)−NEM (2 h)	No	−NEM (▲) +NEM (▲)	No	−NEM (▲) +NEM (▲)	No	−NEM (▲) +NEM (▲)
As(V)−NEM (24 h)	No	−NEM (▲) +NEM (▲)	Yes	−NEM (▼) +NEM (▼)	Yes	−NEM (▼) +NEM (▼)
PQ−BSO (2 h)	No	−BSO (▲) +BSO (▲)	Yes	−BSO (▼) +BSO (▼)	Yes	−BSO (▼) +BSO (▲)
PQ−BSO (24 h)	Yes	−BSO (▲) +BSO (▲)	Yes	−BSO (▼) +BSO (=)	No	−BSO (▼) +BSO (▲)
PQ−NEM (2 h)	No	−NEM (▲) +NEM (▲)	Yes	−NEM (▼) +NEM (▼)	Yes	−NEM (▼) +NEM (▼)
PQ−NEM (24 h)	No	−NEM (▲) +NEM (▲)	Yes	−NEM (▼) +NEM (▼)	No	−NEM (▲) +NEM (▲)
MD−BSO (2 h)	Yes	−BSO (▲) +BSO (▲)	Yes	−BSO (▼) +BSO (▼)	No	−BSO (▼) +BSO (▼)
MD−NEM (2 h)	No	−NEM (▲) +NEM (▲)	No	−NEM (▼) +NEM (▼)	No	−NEM (▲) +NEM (▼)
pH 5−BSO (24 h)	No	−BSO (=) +BSO (=)	Yes	−BSO (▲) +BSO (▼)	No	−BSO (▲) +BSO (▲)
pH 5−NEM (24 h)	No	−NEM (=) +NEM (=)	Yes	−NEM (=) +NEM (=)	No	−NEM (=) +NEM (=)

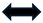
: Interaction. (=): There is no significant difference compared to the control group. (▲): Increase compared to the control. (▼): Decreases compared to the control. ^1^ At the lowest Cd concentration, there is a slight increase, and at the subsequent Cd concentrations, the values remain similar to those from the control.

**Table 2:** Gene expression induction value ranking of TthGCL and TthGR genes in response to the same stressor. Only significant (*p* < 0.05) induction values greater than 2-fold to the control are shown.

Treatment	Fold-Induction Ranking
Cd(II) 2 h	TthGCL > TthGR
Cd(II) 24 h	TthGR > TthGCL
As(V) 2 h	TthGR >> TthGCL
As(V) 24 h	TthGR >> TthGCL
Pb(II) 2 h	TthGR > TthGCL
Pb(II) 24 h	TthGR > TthGCL
Zn(II) 2 h	TthGCL > TthGR
PQ 24 h	TthGCL > TthGR
MD 2 h	TthGCL >> TthGR
CDNB 2 h	TthGR >> TthGCL
pH 5 24 h	TthGCL >> TthGR

## Data Availability

Data will be made available on request.

## Supplementary Materials

The following supporting information can be downloaded at: https://www.mdpi.com/article/10.3390/biology15181616/s1, Figure S1: Alignment of eukaryotic GCL. Figure S2: Alignment of selected eukaryotic GS. Table S1: Primers used in the qRT-PCR analysis. Table S2: Quantitative RT-PCR standard-curve parameters. Table S3: Relative CMFDA fluorescence values (thiol groups) and cell death percentages. Statistical analysis of the results for both parameters. Table S4: Enzymatic determination of total glutathione throughout the growth curve of *T. thermophila*. Table S5: Total glutathione (TG) levels determined enzymatically, in the presence or absence of BSO or NEM and under stress induced by various stressors. Table S6: GR structural features from selected ciliates. Tables S7 and S8: Relative fold-induction values of TthGCL or TthGR gene expression under different stress conditions. Table S9: No interaction. Table S10: Interaction. Text S1: Examples that help explain the statistical analysis and whether or not there is an interaction between the two factors used (inhibitors and stressors).
