# Supplementary material for: Glutathione Relevance in the Response of Ciliate Tetrahymena thermophila Against Metals/Metalloids and Other Environmental Stressors
Source: Biology (Basel). 2026 Sep 14;15(18):1616. doi: 10.3390/biology15181616 (PMC13604404; doi:10.3390/biology15181616)
Supplement: Supplementary file 1 [file biology-15-01616-s001.zip › biology-4475360-supplementary.pdf]

Supplementary Material - Biology

Glutathione relevance in the response of ciliate Tetrahymena thermophila against metals/metalloids and other environmental stressors.

Ruth Ortega, Ana Martin-González and Juan-Carlos Gutiérrez

Figure S1. Alignment of eukaryotic GCL catalytic domain partial sequences. Cysteine-binding residues are shown in red (arrow). Identical amino acids are highlighted in yellow.

```
            ....|....| ....|....| ....|....| ....|....| ....|....|
                   710        720        730        740        750
TthGCL      ---------S KYIPKSRYDS ISYFISDE-- ----KRNLPQ YNDLKFPLNE
TboGCL      ---------D KFIPKSRYDS ISYFISDE-- ----PRNLPQ YNDLKFPLNQ
TelGCL      ---------S KYIPKSRYDS ISLFISDE-- ----KRNLPQ YNDLKFPLNE
TmaGCL      ---------S KYIPKSRYDS ISFYISDE-- ----KRNLPQ YNDLKFPLNE
PbiGCL      ---------P KYIPKSRYST ISYFISND-- ----KMNLEE YNDLNFPANE
PbuGCL      ---------P SYIPKSRYSG ISYFISND-- ----PKNLEE YNDTPFPVNQ
PcaGCL      ---------P RYIPKSRYST ISYYISND-- ----PMNLPE YNDLNFPANE
PteGCL      ---------P NHIPKSRYSS ISYFISND-- ----KMNLEE YNDLNFPANE
EvaGCL      ---------H NYMPKSRYAG MNHYLSDH-- ----PYFCGD GINDGYKLKI
EaeGCL1     ---------E NFVPKSRYST LNHYISDH-- ----QFFSDE ELNDGIKLKI
EaeGCL2     ---------E NFVPKSRYSS MNHYISDH-- ----VYFSNE NLNDGIKLKI
SleGCL      ---------E NYIPKSRYGT QNHYLSNH-- ----KYVKQE YMDT-IQYKV
PcarGCL     ---------V RHVPKTRFSS TNHYISNH-- ----QYVKQD YFDI-YQYNV
ScoGCL1     ---------- ---IFPRYSP SALYLSQC-- ----VTNRPE YNDIPVTIHE
ScoGCL2     ---------- ---VLPRCSP SALYLSQC-- ----ITNKSE YNDVSVNIHQ
SpyGCL      ---------- ---ITSRYGP SELYISQC-- ----QTNKCE YNDVDVHIHQ
OtrGCL1     ---------E QYIPKSRYSS QNHYLSSH-- ----KYVKDQ YIDT-IQYKV
OtrGCL2     ---------E NYIPKSRYST INHYISDH-- ----QYVKQR YMDT-YQYKV
BstGCL      ---------- ---WRSRYGG VSRYISQC-- ----AKLKPE YNDINVPYHA
ImuGCL      ---------K SYIPKSRYGS ISLFISEE-- ----QRNLQI YNDLNFPLNQ
AfuGCL      ---------- WRIPKSRYAS NSTYISQD-- ----PRLRKE YLDPELIVDE
CelGCL      ---------- WVIDKSRYDS TDCYIYPC-- -------SVG YNDIPLQYDE
DmeGCL      ---------- FRIAKSRYDS IDSYLSPE-- -------GAK YNDVPLTYDE
DreGCL      ---------- FRIHKSRYDS IDSYLSCC-- -------GEK YNDIELTIDE
MmuGCL      ---------- FRISKSRYDS IDSYLSKC-- -------GEK YNDIDLTIDK
SceGCL      ---------V LEIPKSRYSS VDLFLGG--- ---SKFFNRT YNDTNVPINE
CalGCL      DLYTKDGKPI QRVPQSRYDS IDNYLNDNYY --DTKYFQDE YNDLNAPINE
DdiGCL      ---------- FVINKSRYDS IDSYIGSK-- ---SKSFRSE YNDLDLVYDK
PcacGCL     ---------V KRLPKSRYEG ISTFICNHKQ GEDPHTSTDK YNDVDVPYDE
EcaGCL      ---------- YRISKSRYDS IDSYLSEC-- -------GEK YNDIDLTIDK
HsaGCL      ---------- YRISKSRYDS IDSYLSKC-- -------GEK YNDIDLTIDK
```

```
            ....|....| ....|....| ....|....| ....|....| ....|....|
                   810        820        830        840        850
TthGCL      EKETLHFENI QSTNWNSVRL KPPPSMDS-- ----NIGWRT EFRTMECQIT
TboGCL      SKETLHFENI QSTNWNSVRL KPPPSMNS-- ----RIGWRF EFRTMECQIS
TelGCL      EKETLHFENI QSTNWNSVRL KPPPSMDS-- ----NIGWRT EFRTMESQIT
TmaGCL      EKETLHFENI QSTNWNSVRL KPPPSMDS-- ----NIGWRT EFRTMECQIT
PbiGCL      N--TAHFEAI QSSNWNSVRF KPPPSYNS-- ----KIGWRV EFRTMEIQLT
PbuGCL      EEVTSHFEAI QSSNWNSVRF KPPPSYNS-- ----KIGWRV EFRTMEIQLT
PcaGCL      S--TAHFEAI QSSNWNSVRF KPPPSYNS-- ----RIGWRV EFRTMEIQLT
```

```
PteGCL      N--TAHFEAI QSSNWNSVRF KPPPSYNS-- ----KIGWRV EFRTMEIQLT
EvaGCL      PDSTEHFENL NSTNWNSVRF KPPPSYDS-- ----PIGWRV EFRPVDIQTT
EaeGCL1     EETTEHFESL NSTNWNSVRF KPPPALDS-- ----SIGWRV EFRTFDIQIT
EaeGCL2     ENSTEHFENF NSTNWNSVRF KPPPSLDS-- ----NIGWRV EFRTLDVQIS
SleGCL      QEITAHFENL QSTNWNSMRF KPPPSVQS-- ----KIGWRV EFRTMDIQIT
PcarGCL     NVLLSHFENL QSTNWNSLRF KPPPALDS-- ----EIGWRI EFRIMDIQLT
ScoGCL1     EKVTNHFENF QSTNWNSVRF KPPPTMDS-- ----NVGWRV EFRTMDIQLS
ScoGCL2     MSTTNHFENF QSTNWNSVRF KPPPNMNS-- ----NVGWRV EFRTMDIQLS
SpyGCL      SLNTNHFENF QSTNWNSVRF KPPPSMDS-- ----TIGWRV EFRSMDIQLT
OtrGCL1     EQTTGHFENL QSTNWCSMRF KPPPSLDS-- ----KIGWRV EFRTMDIQLT
OtrGCL2     SQTTAHFENL QSTNWCSMRF KPPPSKDS-- ----KIGWRV ELRTMDIQLT
BstGCL      TTTTMHFENL QSTNWNSLRF KPPPSMDS-- ----DICWRV EFRPMDVQIT
ImuGCL      SKSTSHFESI QSTNWNSVRI KPPPAMDS-- ----KIGWRV EFRTMECQIT
AfuGCL      LNKADHFENL QSTNWQHMRF KPPPPDK--- ---ADIGWRV EFRSMEIQMT
CelGCL      EKSSEHFETI QSSNWMNMRF KPPPPDAP-- ----EIGWRV EFRPTEVQLT
DmeGCL      NEDTDHFENI QSTNWQTMRF KPPPPN-S-- ----SIGWRV EFRPCEAQIS
DreGCL      ENESDHFENI QSTNWQTMRF KPPPPN-S-- ----EIGWRV EFRPMEVQLT
MmuGCL      ANESDHFENI QSTNWQTMRF KPPPPN-S-- ----DIGWRV EFRPMEVQLT
SceGCL      KTSSNHFENI QSTNWQTLRF KPPTQQATP- DKKDSPGWRV EFRPFEVQLL
CalGCL      NLENDHFENI QSTNWQTLRF KPPALYTKDT DLTTKPGWRV EFRPMEIQLT
DdiGCL      EKNTDHFENI QSTNWQTVRF KPPPP----- --SSSIGWRV ELRPMEVQLT
PcacGCL     EHETDHFENI QSTNWQTVRW KPPPPAPSS- AEGPHIGWRT EFRSMEIQLT
EcaGCL      ANESDHFENI QSTNWQTMRF KPPPPN-S-- ----DIGWRV EFRPMEVQLT
HsaGCL      ANESDHFENI QSTNWQTMRF KPPPPN-S-- ----DIGWRV EFRPMEVQLT
```

Tth (*Tetrahymena thermophila*); Tbo (*T. borealis*); Tel (*T. elliotti*); Tma (*T. malaccensis*); Pbi (*Paramecium biaurelia*); Pbu (*P. bursaria*); Pca (*P. caudatum*); Pte (*P. tetraurelia*); Eva (*Euplotes vanus*); Eae (*E. aediculatus*); Sle (*Stylonychia lemnae*); Pcar (*Pseudokeropnosis carnea)*; Sco (*Stentor coeruleus*); Spy (*Stentor pyriformis*); Otr (*Oxytricha trifallax*); Bst (*Blepharisma stoltei*); Imu (*Ichthyophthirius multifiliis*); Afu (*Aspergillus fumigatus*); Cel (*Caenorhabditis elegans*); Dme (*Drosophila melanogaster*); Dre (*Danio rerio*); Mmu (*Mus musculus*); Sce (*Saccharomyces cerevisiae*); Cal (*Candida albicans*); Ddi (*Dictyostelium discoideum*); Pca (*Phytophthora cactorum*); Eca (*Equus caballus*); Hsa (*Homo sapiens*).

Figure S2. Alignment of selected eukaryotic GS. Three flexible, highly conserved loop regions are important for the activity of these enzymes. Substrate attachment loop (S-loop), glycine rich loop (G-loop) and alanine rich loop (A-loop). Identical amino acids are highlighted in yellow.

```
                                  S-loop
            ....|....| ....|....| ....|....| ....|....| ....|....|
                      560        570        580        590        600
TthGS       TMLVE----- -----GDEIA VIYYRTGYSP DHFPN--EEI WKIREKIELT
TboGS       TMFVE----- -----GDEIA VIYYRTGYSP EHFPS--ERI WEIREKIELT
TelGS       TMLVE----- -----GDEIA VIYYRTGYSP DHFPN--EQI WNIREKIELN
TmaGS       TMLVE----- -----GDEIA VIYYRTGYSP DHFPN--EEI WKIREKIELT
PteGS       ILRAF----- -----GQEVA LVYFRTGYTF DQYEN--EEC WSIREMIELS
PcaGS       ILKAF----- -----GKEVA LVYFRTGYTF DQYEN--EEC WNIREMIELS
PmuGS       VLKAF----- -----GQEIS LVYFRTGYTF DQYEN--EQC WEIREMIELS
SleGS       NALSIN---- -----DHEIA FVYYRTGYQL EQYEN--EKD WQTREMLETT
SpyGS       E---LLYK-- -----NLEIS IAYFRSGYAP EHYLS--DEE WKAREKIEIS
BstGS       A---LIYK-- -----DKEIS VVYFRAGYSP DHYPT--ESE WQARELIDTS
ImuGS       RLFVE----- -----NDEIA IVYFRTGYTP EQYPS--EEA WEIREKIELT
```

```
HsaGS      -DRRLFV--- ----DGQEIA VVYFRDGYMP RQYSL---QN WEARLLLERS
MmuGS      -NRRLFM--- ----DDQEVA VVYFRDGYMP SQYNS---QN WEARLMLERS
SceGS      G-KLFIR--- ---DTEQEIA VVYYRTGYTT TDYTS--EKD WEARLFLEKS
CelGS      -RSDLMI--- ----DGRQVA IVYFRAGYSP DHYPST--KE WEARERMELS
DmeGS      -SKELLL--- ----GSQEVA VIYFRAGYEP GHYHSQ--AD WDARYLMETS
AfuGS      PSRPLIYRPP HSPDTQFEVT TVYLRCFYAP TDYTS--ERD WEARTHLERS
DreGS      -SKRLFV--- ----DGHEVA IVYFRNGYMP QNYTSE--QS WEVRLMMERS
NcrGS      PTRQLLYTLP SNPSLVFEVA VVYLRAGYGP GDYPD--QEA WEARYQIERS
AthGS      -DETLIV--- ----GGQAVA VVYFRSGYTP NDHPS--ESE WNARLLIEES
CalGS      --RLYIK--- ---STMDEVS VVYYRSGYAP SDYDVNPEKT WAARLLLEDT
DdiGS      GS-VLIV--- ----DGMEIS VAYYRAGYTP NDYTSSGGDE WKARLLIERS
AcaGS      ---------- ----DGMTVA VTYFRAGYTP NDYPT--QDE WQARELIERS
PcacGS     GKRTLVI--- ----DEREVS VAYFRSAYTP NDYPT--EHE WAGRTLIERS
EcaGS      -DRRLFM--- ----DGQEVA VVYFRDGYMP SQYSQ---QN WEARLLLERS


                                                    G-loop

           ....|....| ....|....| ....|....| ....|....| ....|....|
                  660        670        680        690        700
TthGS      KIKENFATIY DFEDESKH-- --EELLNLAK NEPQHYILKP QREGGDNNIY
TboGS      QIKENFASIY DFEDESKH-- --QELLDLAK NDPDHYILKP QREGGDNNIY
TelGS      QIKENFANIY DFEDETKH-- --QQLIELVK NEPQHYILKP QREGGDNNIY
TmaGS      QIKENFATIY DFEDETKH-- --QELLNLAK NEPQHYILKP QREGGDNNIY
PteGS      LISENYCKIW GFDHEDQH-- --EKLIEMIQ QNPHDYVLKP QREGGGNNYY
PcaGS      IIKENYCRIW GFDNQNEH-- --QQLIDMIQ KNPHDYVLKP QREGGGNNFY
PmuGS      LIKENYCKIW GFDNAAEH-- --NQLIEMIK KNPHDYVLKP QREGGGNNYY
SleGS      IIKQIFKGIW TLEDIEKPES EVQEIIRKAL DNPHDYVIKP QKEGGGNNFY
SpyGS      NLMENITEIY DFS-QVSLDL --IQNIREN- --PKKWVLKP QREGGGNNIY
BstGS      KLADHFTDLW GFDSPLSQEL --LAAIRSN- --PQDYVVKP QREGGGNNFY
ImuGS      AIKENFAEIL DFENEEEQ-- --QNLIIETK KHPEKYVLKP QREGGDNNIY
HsaGS      RLRATFAGLY SLD-VGEEGD --QAIAEALA -APSRFVLKP QREGGGNNLY
MmuGS      RLRATFAGLY SLD-MGEEGD --QAIAEALA -APSHFVLKP QREGGGNNLY
SceGS      SLLKTFVKIY PLDDTKLGRE --GKRLALS- -EPSKYVLKP QREGGGNNVY
CelGS      DIRASFAGMW ALENTDEVTM --KVVAGAQK -HPEAFVLKP QTEGGAALHT
DmeGS      AVGKIFTGLY SLD-DNEAGN --ASYEMALR -TPERFVLKP QREGGGNNVY
AfuGS      RLRETFAPQY DLSSSGRG-- ---RELALNP ETAVNHVLKP QREGGGNNVY
DreGS      QIRATFAGLY TLD-MGEEGD --NTVAMALA -NPDQYVLKP QREGGGNNIY
NcrGS      RLLKTFTNIY PLDTSSSGLH --ARSLATSP TQCQKYVMKP QREGGGNNFY
AthGS      KLRKCFAGLW SLD--DSE-- ---IVKQAIE -KPGLFVMKP QREGGGNNIY
CalGS      EVLSTFVAIY SLDDSNEGDT --GRKLAFE- -EPEKFVLKP QREGGGNNVY
DdiGS      RVKRSFTGLY SLSKEDIDMS ---VVKEAIE -SPQNYVMKP QREGGGNNIY
AcaGS      RLRKCFAGLY SLD--EGESE --EAIKDALT -NPDDYVMKP QREGGGNNLY
PcacGS     ALETSFAGLY GLEKDSATIE --DVKKMAIA -NPRSFVLKP QREGGGNNLY
EcaGS      RLRATFAGLY SLD-MGEEGD --QAIAKALA -APSRFVLKP QREGGGNNLY


           ....|....| ....|....| ....|....| ....|....| ....|....|
                  710        720        730        740        750
TthGS      GKNIYPFLEK LTPS-----E RKNYILMEKI IPKPSIT--F MIRNRK---L
TboGS      GQKIYPFLME LSPA-----D RKNYILMEKI IPKPSIT--F MVRNRK---L
TelGS      GKNIYTFLEK ITPQ-----E RKNYILMEKI IPKPQIT--F MVRNRK---L
TmaGS      GKNIYPFLEK LTPS-----E RKNYILMEKI IPKPSIT--F MIRNRK---L
PteGS      DDQIIPELLK LTPE-----Q RTEFIVMERI KPIPRIG--F MMRRGQ---L
PcaGS      DDQILPELEK LSPG-----Q RNEYIVMERI KPIPRIG--F MMRRGQ---L
PmuGS      DDQIIPELLK LTPE-----Q RTEYIIMERI KPIPRIG--F MMRRGQ---L
SleGS      DDQVKELLEQ NDKE-----Y LKQFLIMERI NPPEIKA--F MLRNGK---L
SpyGS      GDDILSMLDN YTEH------ -KEYILMKMI ECKEQDT--W MIKASK---A
BstGS      GEGVVKILEN YENDQNIQEH MKQYILMKRI NPEPKEN--L LSRKGV---V
ImuGS      GEKISTFLEK ITVQ-----E RKNYILMEKI VPQPHIC--L MVRNRE---I
```

```
HsaGS        GEEMVQALKQ LKD----SEE RASYILMEKI EPEPFEN--C LLRPGS--PA
MmuGS        GEEMVQALEQ LKD----SEE RASYILMEKI EPEPFRN--C LLRPGS--PA
SceGS        KENIPNFLKG IEER-----H WDAYILMELI EPELNENN-I ILRDNK---S
CelGS        GDEMVQMLRE LP-----EEE RGAFILMEKL KPMIIEN--Y LVLAKK--PI
DmeGS        GVDIPDALKR MS-----RVE RSAWILMDLI HPPLTKG--Y MVRPGGDMPP
AfuGS        KSAIPDFLRS IPES-----E WKRWILMELI RPPAEAKNVA LRSDGE---V
DreGS        GSEICEVLEK LKN----SSE RTAYILMDKI QPVPVQN--I LLRPGA--PL
NcrGS        RSAIPEQLAK IPEE-----H WNSFILMEII EPPALAN--S ILRNGK---V
AthGS        GDDVRENLLR LQKEG--EEG NAAYILMQRI FPKVSNM--F LVREGV---Y
CalGS        KENIPSFLEK LDKQ-----E WGAYILMELI NPPTHKN--K IIRNNE---V
DdiGS        NDQVAIALKS MS-----SEE LSSYILMDKI MSKSFKT--H VVRDRQ---L
AcaGS        GEKLKHALTT MS-----AQE RAAHILMQRI KPQPYEA--Y VYRRGE---S
PcacGS       GDEVASAIQK MS-----PAE LESYILMERI FPKENPA--V LLRNSL---T
EcaGS        GEEMVQALEQ LKD----SEE RASYILMEKI EPEPFGN--C LLRPGS--PT
```

A-loop

```
             ....|....| ....|....| ....|....| ....|....| ....|....|
                     760        770        780        790        800
TthGS        EVAPSISELG IISYFIS--- --DSQKIIHN KAGGYLVRTK RYFDDEGGVN
TboGS        EVAPSISELG IISYFIS--- --DSQKILHN KAGGYLVRTK RYFDNEGGVN
TelGS        EVAPSISELG IISYFIS--- --DKDKIIHN KPGGYLIRSK RYFDDEGGVN
TmaGS        EVAPSISELG IISYFIS--- --DSQKIIHN KAGGYLVRTK RYFDDEGGVN
PteGS        DIQAVISEIS VIGYFIN--- --EGENILVN EVGGYLVRTK RYLDNEGGVA
PcaGS        DVQAVISEIS VIGYFIN--- --EGENIITN QVGGYLVRTK RYLDNEGGVA
PmuGS        DLQAVISEIS VIGYFIN--- --EGDKILCN EVGGYLVRTK RYLDNEGGVA
SleGS        IEGQTLSEFG VFSSIFISTQ HKDQHDVVSQ KIIGRLLRTK GKDSNEGGVN
SpyGS        QVVRSLSEVG MFGTVLAKG- --EE--IIVN EYAGYLVRTK SKESNEGGVA
BstGS        NRVQTLSELG MFGVLVANG- --EE--IVTN VYGGYLLRTK SKDANEGGVA
ImuGS        EAQACVAELG IMSYFIF--- --EGNNIIQS KQGGYLVRSK KYFDDEGGVN
HsaGS        RVVQCISELG IFGVYVRQE- --KT-LVMN- KHVGHLLRTK AIEHADGGVA
MmuGS        QVVQCISELG IFGVYVRQG- --TT-LVMN- KHVGHLLRTK AVEHADGGVA
SceGS        YNEPIISELG IYGCVLFND- --EQ--VLSN EFSGSLLRSK FNTSNEGGVA
CelGS        TFAKAVSELG VYGYAFGR-- --KD-APEL- KTAGHLLRTK PESTAMGGVA
DmeGS        QIVDMVSELG IFGVVIGDA- --EH-IVHN- YQAGHMLRTK LSTANEGGVA
AfuGS        LSGKVIGELG IYGTILWDQ- --SNGNVLQN EQGGWLMRTK AKDVNEGGVA
DreGS        KVSSCLSELG AFGAYVRKG- --SE-LVLN- ECVGHLLRTK SSEHADGGVA
NcrGS        EVGGVICELG VYGTCLWDQ- --SSGEIKFN EEAGYLLRTK GDKSEEGGVA
AthGS        HKHQAISELG VYGAYLRSK- --D--KVIVN EQSGYLMRTK IASSDEGGVA
CalGS        FHEDIISELG VFGTTVFNE- --KTGEILTN KNAGWLLRSK FSNSNEGGVA
DdiGS        LEIEGLYELG IYSVFISNG- --DD-DIVLN KQAGILLRTK TANSDEVGVA
AcaGS        SLVQAICELG VFSVYVSKG- --D--EEFIN EDGGHLLRTK TASSDEGGVA
PcacGS       SSGPTISELG MFITSLFDG- --EG-KEIVN KHAGHLLRTK LSGTDEGGVA
EcaGS        RVDQCISELG IFGVYVRQG- --KT-LVMN- KHVGHLLRTK AIVHADGGVA
```

```
             ....|....| ....|....| ....|....
                     810        820
TthGS        AGYSVIDGLM IKKQQD---- ---------
TboGS        AGYSVIDGLM VKKQQN---- ---------
TelGS        AGYSVIDGLM VKK------- ---------
TmaGS        AGYSVIDGLM IKKQQ----- ---------
PteGS        AGYAVVDSFM VADS------ ---------
PcaGS        AGYAVVDSFM VQDN------ ---------
PmuGS        AGYAVVDSFM VSDE------ ---------
SleGS        AGFAVIDSPL IVDIDDIKNS AIKPLVSSI
SpyGS        KGYAVID--- ---------- ---------
BstGS        AGYGVLDSIY LD-------- ---------
ImuGS        AGHSVIDGVM FKI------- ---------
HsaGS        AGVAVLDNPY PV-------- ---------
MmuGS        AGVAVLDNPY PV-------- ---------
```

```
SceGS        AGFGCLDSII LY-------- ---------
CelGS        AGHAVVDTPF LYEFI----- ---------
DmeGS        AGLGALDSPY LIDSDDEDEQ K--------
AfuGS        TGFSSLDSIL LY-------- ---------
DreGS        AGVAVLDNPL LV-------- ---------
NcrGS        AGYGCMDSVT LV-------- ---------
AthGS        AGFGVLDSIY LN-------- ---------
CalGS        AGFGCVDNIY LY-------- ---------
VcoGS        KLMDAIERRL KATAM----- ---------
PaeGS        KLMDAIAAQL AAR------- ---------
DdiGS        AGFGLLDSPI LE-------- ---------
AcaGS        AGFAVLDSPY LSAW------ ---------
PcacGS       TGFSVVSSPL LI-------- ---------
EcaGS        AGVAVLDNPY PV-------- ---------
```

Tth (*Tetrahymena thermophila*); Tbo (*T. borealis*); Tel (*T. elliotti*); Tma (*T. malaccensis*); Pca (*P. caudatum*); Pte (*P. tetraurelia*); Pmu (*P. multimicronucleatum*); Sle (*Stylonychia lemnae*); Spy (*Stentor pyriformis*); Bst (*Blepharisma stoltei*); Imu (*Ichthyophthirius multifiliis*); Afu (*Aspergillus fumigatus*); Cel (*Caenorhabditis elegans*); Dme (*Drosophila melanogaster*); Dre (*Danio rerio*); Mmu (*Mus musculus*); Sce (*Saccharomyces cerevisiae*); Ncr (*Neurospora crassa*); Ath (*Arabidopsis thaliana*); Cal (*Candida albicans*); Ddi (*Dictyostelium discoideum*); Pcac (*Phytophthora cactorum*); Eca (*Equus caballus*); Hsa (*Homo sapiens*).

Table S1. Primers used in the qRT-PCR analysis.

**Target gene**	**Oligonucleotide name**	**Sequence (5' → 3')**
TthATUB	TthATUB1	TGTCGTCCCCAAGGAT
	TthATUB2	GTTCTCTTGGTCTTGATGGT
TthGCL	TthGCL1	AGAATTCAGAACTATGGAATGCTA
	TthGCL2	ATGAAAGCAGCGTTCTCATC
TthGR	TthGR1	ACTTAGACAGGTGAAC
	TthGR2	ACTCAAGGTTAACGCC

Table S2. Quantitative RT-PCR standard-curve parameters. Amplification efficiency (E), slope (S), correlation coefficient (R^2).

Gene	E (%)(2)	S(1)	R^2
TthATUB	1.94 (104.51)	-3.47	1.00
TthGCL	1.90 (108.43)	-3.60	0.99
TthGR	1.97 (102.40)	-3.40	1.00

(1) A slope of - 3.32 indicates 100% amplification efficiency. (2) A good reaction should have an E value between 90 – 110%.

Table S3. Relative CMFDA fluorescence values (thiol groups) and cell death percentages (Figs. 1-4). Statistical analysis of the results for both parameters. Significant values ($p < 0.05$) are highlighted in yellow (n = 3). For a better interpretation of these data, see supplementary Text S1.

Table S3A. Cd(II)-BSO (2h) (see Fig. 1A)

Thiol groups (CMFDA)

	**Relative fluorescence**			
**Cd(II)**	**-BSO**		**+BSO**	
**(μM)**	**AV**	**SD**	**AV**	**SD**
**0**	1.00	0.00	0.62	0.02
**1.32**	2.11	0.12	1.04	0.05
**1.54**	1.95	0.05	0.91	0.12
**1.76**	1.77	0.07	0.89	0.07

AV: average value. SD: standard deviation.

Death percentages (PI)

	**Cell death percentage**			
**Cd(II)**	**-BSO**		**+BSO**	
**(μM)**	**AV**	**SD**	**AV**	**SD**
**0**	5.75	0.85	3.01	0.76
**1.32**	4.75	0.89	4.41	1.02
**1.54**	4.08	2.79	4.33	1.71
**1.76**	4.47	1.55	4.44	1.93

AV: average value. SD: standard deviation.

CMFDA statistics

**Interaction**	**Exists** (0.000)		
**BSO effect**			
**Cd(II)** (0): (0.000)	Cd(II) (1.32): (0.001)	Cd(II) (1.54): (0.002)	Cd(II) (1.76): (0.000)
**Cd(II) effect**			
**BSO (-)**	0 - 1.32: (0.004)	1.32 - 1.54: (0.013)	1.54 – 1.76: (0.048)
**BSO (+)**	0 – 1.32: (0.001)	1.32 – 1.54: (0.090)	1.54 - 1.76: (0.539)

PI statistics

**Interaction**	**Does not exist** (0.360)		
**BSO effect**	(0.364)		
**Cd(II) effect**	0 – 1.32 (0.763)	1.32 – 1.54 (0.708)	1.54 – 1.76 (0.539)

Table S3B. Cd(II)-BSO (24h) (see Fig. 1B)

Thiol groups (CMFDA)

Cd(II)	Relative fluorescence			
	-BSO		+BSO	
(μM)	AV	SD	AV	SD
**0**	1.00	0.00	0.24	0.00
**1.32**	1.85	0.11	0.69	0.07
**1.54**	2.05	0.05	0.74	0.07
**1.76**	2.24	0.16	0.76	0.01

AV: average value. SD: standard deviation.

Death percentages (PI)

Cd(II)	Cell death percentage			
	-BSO		+BSO	
(μM)	AV	SD	AV	SD
**0**	6.41	2.55	3.01	0.76
**1.32**	8.79	3.24	5.98	1.34
**1.54**	19.02	2.07	18.36	0.37
**1.76**	93.14	2.82	45.89	7.75

AV: average value. SD: standard deviation.

CMFDA statistics

**Interaction**	**Exists** (0.011)		
**BSO effect**			
**Cd(II)** (0): (0.000)	Cd(II) (1.32): (0.000)	Cd(II) (1.54): (0.000)	Cd(II) (1.76): (0.004)
**Cd(II) effect**			
**BSO (-)**	0 - 1.32: (0.006)	1.32 - 1.54: (0.025)	1.54 – 1.76: (0.218)
**BSO (+)**	0 – 1.32: (0.027)	1.32 – 1.54: (0.137)	1.54 - 1.76: (0.435)

PI statistics

**Interaction**	**Exists** (0.000)		
**BSO effect**			
**Cd(II)** (0): (0.093)	Cd(II) (1.32): (0.238)	Cd(II) (1.54): (0.614)	Cd(II) (1.76): (0.001)
**Cd(II) effect**			
**BSO (-)**	0 - 1.32: (0.283)	1.32 - 1.54: (0.079)	1.54 – 1.76: (0.001)
**BSO (+)**	0 – 1.32: (0.069)	1.32 – 1.54: (0.002)	1.54 - 1.76: (0.023)

Table S3C. Cd(II)-NEM (2h) (see Fig. 1C)

Thiol groups (CMFDA)

	**Relative fluorescence**			
**Cd(II)**	**-NEM**		**+NEM**	
**(μM)**	**AV**	**SD**	**AV**	**SD**
**0**	1.00	0.00	0.80	0.07
**1.32**	1.34	0.15	0.79	0.17
**1.54**	1.15	0.06	0.68	0.01
**1.76**	1.10	0.06	0.64	0.25

AV: average value. SD: standard deviation.

Death percentages (PI)

	**Cell death percentage**			
**Cd(II)**	**-NEM**		**+NEM**	
**(μM)**	**AV**	**SD**	**AV**	**SD**
**0**	1.55	0.59	2.37	1.13
**1.32**	3.79	1.56	2.72	1.31
**1.54**	2.92	1.65	3.35	2.18
**1.76**	2.93	2.36	4.55	3.04

AV: average value. SD: standard deviation.

CMFDA statistics

**Interaction**	**Does not exist** (0.178)		
**NEM effect**	(0.003)		
**Cd(II) effect**	0 – 1.32 (0.074)	1.32 – 1.54 (0.034)	1.54 – 1.76 (0.712)

PI statistics

**Interaction**	**Does not exist (0.587)**		
**NEM effect**	(0.559)		
**Cd(II) effect**	0 – 1.32 (0.124)	1.32 – 1.54 (0.838)	1.54 – 1.76 (0.712)

Table S3D. Cd(II)-NEM (24h) (see Fig. 1D)

Thiol groups (CMFDA)

	**Relative fluorescence**			
**Cd(II)**	**-NEM**		**+NEM**	
**(μM)**	**AV**	**SD**	**AV**	**SD**
**0**	1.00	0.00	0.30	0.05
**1.32**	2.08	0.27	0.38	0.01
**1.54**	2.07	0.32	0.39	0.02
**1.76**	1.97	0.15	0.34	0.12

AV: average value. SD: standard deviation.

Death percentages (PI)

	**Cell death percentage**			
**Cd(II)**	**-NEM**		**+NEM**	
**(μM)**	**AV**	**SD**	**AV**	**SD**
**0**	3.75	1.46	6.97	3.53
**1.32**	4.05	1.63	15.57	1.96
**1.54**	10.10	3.12	65.87	3.76
**1.76**	91.54	3.56	92.05	5.57

AV: average value. SD: standard deviation.

CMFDA statistics

**Interaction**	**Exists** (0.000)		
**NEM effect**			
**Cd(II)** (0): (0.000)	Cd(II) (1.32): (0.000)	Cd(II) (1.54): (0.000)	Cd(II) (1.76): (0.001)
**Cd(II) effect**			
**NEM (-)**	0 - 1.32: (0.001)	1.32 - 1.54: (0.864)	1.54 – 1.76: (0.191)
**NEM (+)**	0 – 1.32: (0.030)	1.32 – 1.54: (0.329)	1.54 - 1.76: (0.353)

PI statistics

**Interaction**	**Exists** (0.000)		
**NEM effect**			
**Cd(II)** (0): (0.215)	Cd(II) (1.32): (0.001)	Cd(II) (1.54): (0.000)	Cd(II) (1.76): (0.900)
**Cd(II) effect**			
**NEM (-)**	0 - 1.32: (0.849)	1.32 - 1.54: (0.086)	1.54 – 1.76: (0.001)
**NEM (+)**	0 – 1.32: (0.113)	1.32 – 1.54: (0.001)	1.54 - 1.76: (0.0.39)

Table S3E. As(V)-BSO (2h) (see Fig. 2A)

Thiol groups (CMFDA)

	Relative fluorescence			
**As(V)**	**-BSO**		**+BSO**	
**(μM)**	**AV**	**SD**	**AV**	**SD**
**0**	1.00	0.00	0.75	0.11
**33**	2.14	0.13	1.42	0.36
**66**	1.91	0.12	1.19	0.16
**133**	1.63	0.07	1.09	0.13

AV: average value. SD: standard deviation.

Death percentages (PI)

	Cell death percentage			
**As(V)**	**-BSO**		**+BSO**	
**(μM)**	**AV**	**SD**	**AV**	**SD**
**0**	4.96	3.21	5.97	1.81
**33**	4.97	0.62	3.80	0.95
**66**	7.69	1.45	6.52	1.14
**133**	5.36	3.73	7.43	3.33

AV: average value. SD: standard deviation.

CMFDA statistics

**Interaction**	**Exists** (0.040)		
**BSO effect**			
**As(V)** (0): (0.019)	As(V) (33): (0.039)	As(V) (66): (0.004)	As(V) (133): (0.003)
**As(V) effect**			
**BSO (-)**	0 - 33: (0.004)	33 - 66: (0.001)	66 – 133: (0.010)
**BSO (+)**	0 – 33: (0.043)	33 – 66: (0.173)	66 - 133: (0.024)

PI statistics

**Interaction**	**Does not exist** (0.506)		
**BSO effect**	(0.838)		
**As(V) effect**	0 – 33 (0.366)	33 – 66 (0.009)	66 – 133 (0.726)

Table S3F. As(V)-BSO (24h) (see Fig. 2B)

Thiol groups (CMFDA)

	Relative fluorescence			
**As(V)**	**-BSO**		**+BSO**	
**(μM)**	**AV**	**SD**	**AV**	**SD**
**0**	1.00	0.00	0.37	0.05
**33**	0.75	0.11	0.25	0.03
**66**	0.64	0.11	0.23	0.03
**133**	0.43	0.02	0.14	0.06

AV: average value. SD: standard deviation.

Death percentages (PI)

	Cell death percentage			
**As(V)**	**-BSO**		**+BSO**	
**(μM)**	**AV**	**SD**	**AV**	**SD**
**0**	6.3	1.17	5.65	1.32
**33**	19.19	2.81	19.17	3.82
**66**	47.92	8.30	46.87	5.70
**133**	89.90	4.62	91.82	6.48

AV: average value. SD: standard deviation.

CMFDA statistics

**Interaction**	**Exists** (0.011)		
**BSO effect**			
**As(V)** (0): (0.000)	As(V) (33): (0.002)	As(V) (66): (0.003)	As(V) (133): (0.001)
**As(V) effect**			
**BSO (-)**	0 - 33: (0.047)	33 - 66: (0.001)	66 – 133: (0.122)
**BSO (+)**	0 – 33: (0.073)	33 – 66: (0.020)	66 - 133: (0.279)

PI statistics

**Interaction**	**Does not exist** (0.888)		
**BSO effect**	(0.797)		
**As(V) effect**	0 – 33 (0.002)	33 – 66 (0.001)	66 – 133 (0.000)

Table S3G. As(V)-NEM (2h) (see Fig. 2C)

Thiol groups (CMFDA)

	**Relative fluorescence**			
**As(V)**	**-NEM**		**+NEM**	
**(μM)**	**AV**	**SD**	**AV**	**SD**
**0**	1.00	0.00	0.84	0.08
**33**	1.64	0.08	1.54	0.38
**66**	1.54	0.22	1.07	0.27
**133**	0.90	0.19	0.75	0.13

AV: average value. SD: standard deviation.

Death percentages (PI)

	**Cell death percentage**			
**As(V)**	**-NEM**		**+NEM**	
**(μM)**	**AV**	**SD**	**AV**	**SD**
**0**	2.32	0.94	5.46	0.77
**33**	5.13	2.47	7.86	2.21
**66**	5.34	0.86	6.51	1.52
**133**	5.59	2.99	6.43	3.34

AV: average value. SD: standard deviation.

CMFDA statistics

**Interaction**	**Does not exist** (0.418)		
**NEM effect**	(0.040)		
**As(V) effect**	0 – 33 (0.002)	33 – 66 (0.006)	66 – 133 (0.099)

PI statistics

**Interaction**	**Does not exist** (0.668)		
**NEM effect**	(0.097)		
**As(V) effect**	0 – 33 (0.105)	33 – 66 (0.655)	66 – 133 (0.940)

Table S3H. As(V)-NEM (24h) (see Fig. 2D)

Thiol groups (CMFDA)

	Relative fluorescence			
**As(V)**	**-NEM**		**+NEM**	
**(μM)**	**AV**	**SD**	**AV**	**SD**
**0**	1.00	0.00	0.77	0.02
**33**	0.87	0.10	0.64	0.05
**66**	0.44	0.05	0.34	0.00
**133**	0.27	0.02	0.22	0.02

AV: average value. SD: standard deviation.

Death percentages (PI)

	Cell death percentage			
**As(V)**	**-NEM**		**+NEM**	
**(μM)**	**AV**	**SD**	**AV**	**SD**
**0**	5.26	2.12	7.49	2.19
**33**	15.66	6.46	22.43	2.85
**66**	54.47	12.02	61.58	5.87
**133**	88.73	2.17	93.21	4.64

AV: average value. SD: standard deviation.

CMFDA statistics

**Interaction**	**Exists** (0.027)		
**NEM effect**			
**As(V)** (0): (0.000)	As(V) (33): (0.034)	As(V) (66): (0.055)	As(V) (133): (0.025)
**As(V) effect**			

**NEM (-)**	0 - 33: (0.148)	33 - 66: (0.004)	66 – 133: (0.020)
**NEM (+)**	0 – 33: (0.034)	33 – 66: (0.009)	66 - 133: (0.002)

PI statistics

**Interaction**	**Does not exist** (0.692)		
**NEM effect**	(0.060)		
**As(V) effect**	0 – 33 (0.002)	33 – 66 (0.001)	66 – 133 (0.002)

Table S3I. PQ-BSO (2h) (see Fig. 3A)

Thiol groups (CMFDA)

	Relative fluorescence			
**PQ**	**-BSO**		**+BSO**	
**(mM)**	**AV**	**SD**	**AV**	**SD**
**0**	1.00	0.00	0.65	0.06
**2.5**	0.39	0.11	0.32	0.11
**5**	0.32	0.12	0.28	0.11
**10**	0.32	0.09	0.26	0.12

AV: average value. SD: standard deviation.

Death percentages (PI)

	Cell death percentage			
**PQ**	**-BSO**		**+BSO**	
**(mM)**	**AV**	**SD**	**AV**	**SD**
**0**	1.68	1.20	1.57	1.50
**2.5**	3.18	1.50	1.72	0.63
**5**	2.57	0.83	2.60	1.58
**10**	5.32	0.69	3.07	1.90

AV: average value. SD: standard deviation.

CMFDA statistics

**Interaction**	**Exists** (0.016)		
**BSO effect**			
**PQ** (0): (0.011)	PQ (2.5): (0.510)	PQ (5): (0.618)	PQ (10): (0.585)
**PQ effect**			
**BSO (-)**	0 – 2.5: (0.013)	2.5 - 5: (0.011)	5 – 10: (0.525)

**BSO (+)**	0 – 2.5: (0.012)	2.5 – 5: (0.020)	5 - 10: (0.199)

PI statistics

**Interaction**	**Does not exist** (0.320)		
**BSO effect**	(0.225)		
**PQ effect**	0 – 2.5 (0.179)	2.5 – 5 (0.822)	5 – 10 (0.000)

Table S3J. PQ-BSO (24h) (see Fig. 3B)

Thiol groups (CMFDA)

	**Relative fluorescence**			
**PQ**	**-BSO**		**+BSO**	
**(mM)**	**AV**	**SD**	**AV**	**SD**
**0**	1.00	0.00	0.13	0.03
**2.5**	0.64	0.41	0.08	0.01
**5**	0.43	0.00	0.13	0.03
**10**	0.27	0.05	0.13	0.00

AV: average value. SD: standard deviation.

Death percentages (PI)

	**Cell death percentage**			
**PQ**	**-BSO**		**+BSO**	
**(mM)**	**AV**	**SD**	**AV**	**SD**
**0**	2.31	0.86	2.14	0.71
**2.5**	16.02	2.20	6.93	0.54
**5**	56.51	3.98	20.78	0.69
**10**	82.04	4.07	79.04	5.17

AV: average value. SD: standard deviation.

CMFDA statistics

**Interaction**	**Exists** (0.043)		
**BSO effect**			
**PQ** (0): (0.000)	PQ (2.5): (0.093)	PQ (5): (0.003)	PQ (10): (0.035)
**PQ effect**			
**BSO (-)**	0 – 2.5: (0.505)	2.5 - 5: (0.257)	5 – 10: (0.044)
**BSO (+)**	0 – 2.5: (0.073)	2.5 – 5: (0.120)	5 - 10: (0.547)

PI statistics

**Interaction**	**Exists** (0.001)		
**BSO effect**			
**PQ** (0): (0.809)	PQ (2.5): (0.002)	PQ (5): (0.000)	PQ (10): (0.473)
**PQ effect**			
**BSO (-)**	0 – 2.5: (0.005)	2.5 - 5: (0.006)	5 – 10: (0.018)
**BSO (+)**	0 – 2.5: (0.010)	2.5 – 5: (0.000)	5 - 10: (0.003)

Table S3K. PQ-NEM (2h) (see Fig. 3C)

Thiol groups (CMFDA)

	**Relative fluorescence**			
**PQ**	**-NEM**		**+NEM**	
**(mM)**	**AV**	**SD**	**AV**	**SD**
**0**	1.00	0.00	0.32	0.02
**2.5**	0.41	0.12	0.28	0.06
**5**	0.34	0.05	0.30	0.12
**10**	0.41	0.07	0.34	0.07

AV: average value. SD: standard deviation.

Death percentages (PI)

	**Cell death percentage**			
**PQ**	**-NEM**		**+NEM**	
**(mM)**	**AV**	**SD**	**AV**	**SD**
**0**	1.70	1.36	3.24	0.81
**2.5**	3.17	2.87	4.73	1.98
**5**	2.47	1.00	4.09	1.13
**10**	2.79	1.35	4.25	0.20

AV: average value. SD: standard deviation.

CMFDA statistics

**Interaction**	**Exists** (0.003)		
**NEM effect**			
**PQ** (0): (0.015)	PQ (2.5): (0.413)	PQ (5): (0.574)	PQ (10): (0.693)
**PQ effect**			

**NEM (-)**	0 – 2.5: (0.032)	2.5 - 5: (0.452)	5 – 10: (0.423)
**NEM (+)**	0 – 2.5: (0.456)	2.5 – 5: (0.102)	5 - 10: (0.089)

PI statistics

**Interaction**	**Does not exist** (0.652)		
**NEM effect**	(0.568)		
**PQ effect**	0 – 2.5: (0.302)	2.5 – 5: (0.837)	5 – 10: (0.480)

Table S3L. PQ-NEM (24h) (see Fig. 3D)

Thiol groups (CMFDA)

**PQ (mM)**	**Relative fluorescence**			
	**-NEM**		**+NEM**	
	**AV**	**SD**	**AV**	**SD**
**0**	1.00	0.00	0.36	0.02
**2.5**	0.36	0.26	0.29	0.02
**5**	0.34	0.29	0.22	0.01
**10**	0.25	0.20	0.11	0.03

AV: average value. SD: standard deviation.

Death percentages (PI)

**PQ (mM)**	**Cell death percentage**			
	**-NEM**		**+NEM**	
	**AV**	**SD**	**AV**	**SD**
**0**	2.80	1.54	3.39	1.60
**2.5**	9.63	3.21	17.86	2.23
**5**	53.15	5.03	66.40	4.61
**10**	95.03	6.56	94.03	5.29

AV: average value. SD: standard deviation.

CMFDA statistics

**Interaction**	**Exists (0.032)**		
**NEM effect**			
**PQ** (0): (0.000)	PQ (2.5): (0.438)	PQ (5): (0.336)	PQ (10): (0.182)
**PQ effect**			
**NEM (-)**	0 – 2.5: (0.057)	2.5 - 5: (0.678)	5 – 10: (0.173)

**NEM (+)**	0 – 2.5: (0.000)	2.5 – 5: (0.002)	5 - 10: (0.006)

PI statistics

**Interaction**	**Does not exist** (0.112)		
**NEM effect**	(0.005)		
**PQ effect**	0 – 2.5: (0.003)	2.5 – 5: (0.000)	5 – 10: (0.001)

Table S3M. MD-BSO (2h) (see Fig. 4A)

Thiol groups (CMFDA)

**MD (mM)**	**Relative fluorescence**			
	**-BSO**		**+BSO**	
	**AV**	**SD**	**AV**	**SD**
**0**	1.00	0.00	0.70	0.09
**5**	0.67	0.01	0.51	0.06
**7.5**	0.45	0.02	0.29	0.09
**10**	0.26	0.08	0.22	0.04

AV: average value. SD: standard deviation.

Death percentages (PI)

**MD (mM)**	**Cell death percentage**			
	**-BSO**		**+BSO**	
	**AV**	**SD**	**AV**	**SD**
**0**	1.65	0.50	2.23	0.99
**5**	17.67	0.83	7.63	2.88
**7.5**	57.35	4.74	28.04	1.76
**10**	88.31	8.73	74.90	5.42

AV: average value. SD: standard deviation.

CMFDA statistics

**Interaction**	**Exists** (0.017)		
**BSO effect**			
**MD** (0): (0.004)	MD (5): (0.006)	MD (7.5): (0.045)	MD (10): (0.482)
**MD effect**			
**BSO (-)**	0 – 5: (0.001)	5 – 7.5: (0.008)	7.5 – 10: (0.081)
**BSO (+)**	0 – 5: (0.014)	5 – 7.5: (0.012)	7.5 - 10: (0.119)

PI statistics

Interaction	**Exists** (0.004)		
**BSO effect**			
**MD** (0): (0.414)	MD (5): (0.004)	MD (7.5): (0.001)	MD (10): (0.087)
**MD effect**			
**BSO (-)**	0 – 5: (0.002)	5 – 7.5: (0.004)	7.5 – 10: (0.022)
**BSO (+)**	0 – 5: (0.042)	5 – 7.5: (0.006)	7.5 - 10: (0.119)

Table S3N. MD-NEM (2h) (see Fig. 4B)

Thiol groups (CMFDA)

MD (mM)	Relative fluorescence			
	-NEM		+NEM	
	AV	SD	AV	SD
**0**	1.00	0.00	0.78	0.10
**5**	0.71	0.02	0.58	0.11
**7.5**	0.54	0.10	0.36	0.05
**10**	0.38	0.02	0.31	0.08

AV: average value. SD: standard deviation.

Death percentages (PI)

MD (mM)	Cell death percentage			
	-NEM		+NEM	
	AV	SD	AV	SD
**0**	1.36	1.00	2.03	1.27
**5**	15.41	2.81	20.79	3.05
**7.5**	58.90	4.00	76.67	4.12
**10**	89.12	8.37	88.64	8.31

AV: average value. SD: standard deviation.

CMFDA statistics

Interaction	**Does not exist** (0.091)		
**NEM effect**	(0.052)		
**MD effect**	0 – 5: (0.000)	5 – 7.5: (0.002)	7.5 – 10: (0.009)

PI statistics

**Interaction**	**Does not exist** (0.079)		
**NEM effect**	(0.025)		
**MD effect**	0 – 5: (0.000)	5 – 7.5: (0.000)	7.5 – 10: (0.087)

Table S3O. pH-BSO (24h) (see Fig. 4C)

Thiol groups (CMFDA)

**pH**	**Relative fluorescence**			
	**-BSO**		**+BSO**	
	**AV**	**SD**	**AV**	**SD**
**7**	1.00	0.00	0.33	0.15
**5**	1.31	0.07	0.25	0.19

AV: average value. SD: standard deviation.

Death percentages (PI)

**pH**	**Cell death percentage**			
	**-BSO**		**+BSO**	
	**AV**	**SD**	**AV**	**SD**
**7**	7.97	1.26	6.00	0.66
**5**	7.53	2.50	6.30	3.26

AV: average value. SD: standard deviation.

CMFDA statistics

**Interaction**	**Exists** (0.003)
**BSO effect**	
**pH 7:** (0.021)	pH 5: (0.002)
**pH effect**	
**BSO (-)**	pH 7 – pH 5: (0.031)
**BSO (+)**	pH 7 – pH 5: (0.095)

PI statistics

**Interaction**	**Does not exist** (0.768)
**BSO effect**	(0.299)
**pH effect**	pH 7 – pH 5: (0.957)

Table S3P. pH-NEM (24h) (see Fig. 4D)

Thiol groups (CMFDA)

pH	Relative fluorescence			
	-NEM		+NEM	
	AV	SD	AV	SD
**7**	1.00	0.00	0.12	0.01
**5**	1.02	0.07	0.09	0.02

AV: average value. SD: standard deviation.

Death percentages (PI)

pH	Cell death percentage			
	-NEM		+NEM	
	AV	SD	AV	SD
**7**	6.13	3.12	7.07	2.97
**5**	7.00	1.93	7.37	2.16

AV: average value. SD: standard deviation.

CMFDA statistics

**Interaction**	**Exists** (0.025)
**NEM effect**	
**pH 7:** (0.000)	pH 5: (0.000)
**pH effect**	
**NEM (-)**	pH 7 – pH 5: (0.092)
**NEM (+)**	pH 7 – pH 5: (0.209)

PI statistics

**Interaction**	**Does not exist** (0.583)
**NEM effect**	(0.767)
**pH effect**	pH 7 – pH 5: (0.289)

Table S4. Enzymatic determination of total glutathione throughout the growth curve of *T. thermophila*.

Time (h)	Total glutathione		Cells / mL
	AV	SD	
**0**	3.00	0.13	5 x 10^3
**0.5**	3.01	0.13	5 x 10^3
**5**	3.00	0.12	1 x 10^4
**10**	3.09	0.30	3 x 10^4
**18**	2.95	0.19	5 x 10^4
**24**	3.71	0.19	2 x 10^5
**30**	6.71	0.10	3 x 10^5
**48**	4.70	0.09	3 x 10^5

AV: average value. SD: standard deviation.

Table S5. Total glutathione levels determined enzymatically, in the presence or absence of BSO or NEM and under stress induced by various stressors (Figs. 6-9). Statistical analysis of these results. Significant values ($p < 0.05$) are highlighted in yellow ($n = 3$). For a better interpretation of these data, see supplementary Text S1.

Table S5A. Cd(II)-BSO (2h) (see Fig. 6A)

Glutathione assessment

Cd(II) (μM)	Total glutathione (nmoles)			
	-BSO		+BSO	
	AV	SD	AV	SD
**0**	3.15	0.01	2.81	0.10
**1.32**	3.07	0.15	2.96	0.02
**1.54**	3.26	0.17	2,78	0.15
**1.76**	3.22	0.06	2.90	0.04

AV: average value. SD: standard deviation.

Statistical analysis

**Interaction**	**Does not exist** (0.077)		
**BSO effect**	(0.005)		
**Cd(II) effect**	0 – 1.32: (0.550)	1.32 – 1.54: (0.897)	1.54 – 1.76: (0.603)

Table S5B. Cd(II)-BSO (24h) (see Fig. 6B)

Glutathione assessment

Cd(II) (μM)	Total glutathione (nmoles)			
	-BSO		+BSO	
	AV	SD	AV	SD
**0**	3.18	0.02	2.87	0.07
**1.32**	3.46	0.03	3.29	0.06
**1.54**	3.46	0.06	3.42	0.12
**1.76**	3.88	0.05	3.43	0.01

AV: average value. SD: standard deviation.

Statistical analysis

**Interaction**	**Exists (**0.005**)**		
**BSO effect**			
**Cd(II)** (0): **(**0.001**)**	Cd(II) (1.32): (0.013)	Cd(II) (1.54): (0.253)	Cd(II) (1.76): (0.000)
**Cd(II) effect**			
**BSO (-)**	0 - 1.32: (0.000)	1.32 - 1.54: (0.220)	1.54 – 1.76: (0.010)
**BSO (+)**	0 – 1.32: (0.000)	1.32 – 1.54: (0.056)	1.54 - 1.76: (0.946)

Table S5C. Cd(II)-NEM (2h) (see Fig. 6C)

Glutathione assessment

Cd(II) (μM)	Total glutathione (nmoles)			
	-NEM		+NEM	
	AV	SD	AV	SD
**0**	3.53	0.18	2.94	0.40
**1.32**	4.32	0.04	2.38	0.00
**1.54**	3.62	0.18	2.72	0.32
**1.76**	3.62	0.15	2.72	0.32

AV: average value. SD: standard deviation.

Statistical analysis

**Interaction**	**Exists (**0.006**)**		
**NEM effect**			
**Cd(II)** (0): **(**0.079**)**	Cd(II) (1.32): (0.000)	Cd(II) (1.54): (0.013)	Cd(II) (1.76): (0.012)
**Cd(II) effect**			

**NEM (-)**	0 - 1.32: (0.010)	1.32 - 1.54: (0.014)	1.54 – 1.76: (0.423)
**NEM (+)**	0 – 1.32: (0.136)	1.32 – 1.54: (0.208)	1.54 - 1.76: (0.423)

Table S5D. Cd(II)-NEM (24h) (see Fig. 6D)

Glutathione assessment

Cd(II) (μM)	Total glutathione (nmoles)			
	-NEM		+NEM	
	AV	SD	AV	SD
**0**	3.41	0.11	2.46	0.03
**1.32**	3.46	0.38	2.89	0.23
**1.54**	3.71	0.12	2.77	0.15
**1.76**	3.93	0.09	2.97	0.01

AV: average value. SD: standard deviation.

Statistical analysis

**Interaction**	**Does not exist (0.108)**		
**NEM effect**	(0.002)		
**Cd(II) effect**	0 – 1.32: (0.067)	1.32 – 1.54: (0.719)	1.54 – 1.76: (0.005)

Table S5E. As(V)-BSO (2h) (see Fig. 7A)

Glutathione assessment

As(V) (μM)	Total glutathione (nmoles)			
	-BSO		+BSO	
	AV	SD	AV	SD
**0**	3.27	0.06	2.91	0.05
**33**	3.47	0.23	2.72	0.27
**66**	3.54	0.28	2.69	0.16
**133**	3.87	0.05	2.48	0.05

AV: average value. SD: standard deviation.

Statistical analysis

**Interaction**	**Exists (**0.010)
**BSO effect**	

**As(V)** (0): **(**0.001**)**	As(V) (33): (0.100)	As(V) (66): (0.007)	As(V) (133): (0.000)
**As(V) effect**			
**BSO (-)**	0 - 33: (0.355)	33 - 66: (0.814)	66 - 133: (0.123)
**BSO (+)**	0 – 33: (0.527)	33 - 66: (0.237)	66 - 133: (0.045)

Table S5F. As(V)-BSO (24h) (see Fig. 7B)

Glutathione assessment

As(V) (μM)	Total glutathione (nmoles)			
	-BSO		+BSO	
	AV	SD	AV	SD
**0**	3.82	0.08	3.65	0.07
**33**	3.75	0.31	3.55	0.05
**66**	3.99	0.06	3.54	0.16
**133**	3.79	0.18	3.81	0.13

AV: average value. SD: standard deviation.

Statistical analysis

**Interaction**	**Does not exist (0.161)**		
**BSO effect**	(0.033)		
**As(V) effect**	0 – 33: (0.255)	33 – 66: (0.230)	66 – 133: (0.204)

Table S5G. As(V)-NEM (2h) (see Fig. 7C)

Glutathione assessment

As(V) (μM)	Total glutathione (nmoles)			
	-NEM		+NEM	
	AV	SD	AV	SD
**0**	3.28	0.04	2.88	0.07
**33**	3.71	0.19	3.15	0.02
**66**	3.84	0.07	3.26	0.02
**133**	4.06	0.11	3.45	0.29

AV: average value. SD: standard deviation.

Statistical analysis

**Interaction**	**Does not exist (0.461)**		
**NEM effect**	(0.002)		
**As(V) effect**	0 – 33: (0.008)	33 – 66: (0.163)	66 – 133: (0.026)

Table S5H. As(V)-NEM (24h) (see Fig. 7D)

Glutathione assessment

As(V) (μM)	Total glutathione (nmoles)			
	-NEM		+NEM	
	AV	SD	AV	SD
**0**	3.72	0.09	2.55	0.13
**33**	4.06	0.25	2.34	0.02
**66**	3.11	0.10	2.26	0.04
**133**	2.63	0.07	2.17	0.07

AV: average value. SD: standard deviation.

Statistical analysis

**Interaction**	**Exists** (0.003)		
**NEM effect**			
**As(V)** (0): (0.000)	As(V) (33): (0.007)	As(V) (66): (0.002)	As(V) (133): (0.001)
**As(V) effect**			
**NEM (-)**	0 - 33: (0.067)	33 - 66: (0.043)	66 - 133: (0.020)
**NEM (+)**	0 - 33: (0.100)	33 - 66: (0.038)	66 - 133: (0.042)

Table S5I. PQ-BSO (2h) (see Fig. 8A)

Glutathione assessment

PQ (mM)	Total glutathione (nmoles)			
	-BSO		+BSO	
	AV	SD	AV	SD
**0**	4.11	0.15	2.61	0.17
**2.5**	3.44	0.06	2.84	0.01
**5**	3.34	0.09	3.49	0.03
**10**	3.74	0.14	3.41	0.06

AV: average value. SD: standard deviation.

Statistical analysis

**Interaction**	**Exists** (0.000)		
**BSO effect**			
**PQ** (0): (0.000)	PQ (2.5): (0.003)	PQ (5): (0.058)	PQ (10): (0.017)
**PQ effect**			
**BSO (-)**	0 – 2.5: (0.008)	2.5 - 5: (0.383)	5 - 10: (0.089)
**BSO (+)**	0 – 2.5: (0.060)	2.5 - 5: (0.000)	5 - 10: (0.225)

Table S5J. PQ-BSO (24h) (see Fig. 8B)

Glutathione assessment

	**Total glutathione (nmoles)**			
**PQ**	**-BSO**		**+BSO**	
**(mM)**	**AV**	**SD**	**AV**	**SD**
**0**	4.65	0.17	3.72	0.06
**2.5**	3.97	0.09	3.79	0.06
**5**	3.74	0.12	3.85	0.20
**10**	4.28	0.36	3.81	0.11

AV: average value. SD: standard deviation.

Statistical analysis

**Interaction**	**Does not exist (0.092)**		
**NEM effect**	(0.013)		
**PQ effect**	0 – 2.5: (0.003)	2.5 - 5: (0.528)	5 - 10: (0.728)

Table S5K. PQ-NEM (2h) (see Fig. 8C)

Glutathione assessment

	**Total glutathione (nmoles)**			
**PQ**	**-NEM**		**+NEM**	
**(mM)**	**AV**	**SD**	**AV**	**SD**
**0**	4.65	0.07	4.70	0.40
**2.5**	4.34	0.05	4.59	0.45
**5**	5.00	0.17	3.47	0.12
**10**	3.81	0.02	2.91	0.05

AV: average value. SD: standard deviation.

Statistical analysis

**Interaction**	**Exists** (0.005)		
**NEM effect**			
**PQ** (0): (0.357)	PQ (2.5): (0605)	PQ (5): (0.000)	PQ (10): (0.000)
**PQ effect**			
**NEM (-)**	0 – 2.5: (0.021)	2.5 - 5: (0.024)	5 - 10: (0.011)
**NEM (+)**	0 – 2.5: (0.120)	2.5 - 5: (0.100)	5 - 10: (0.225)

Table S5L. PQ-NEM (24h) (see Fig. 8D)

Glutathione assessment

	Total glutathione (nmoles)			
**PQ**	**-NEM**		**+NEM**	
**(mM)**	**AV**	**SD**	**AV**	**SD**
**0**	4.54	0.02	3.32	0.16
**2.5**	5.17	0.12	3.66	0.09
**5**	4.52	0.32	3.77	0.21
**10**	4.37	0.08	3.37	0.08

AV: average value. SD: standard deviation.

Statistical analysis

**Interaction**	**Does not exist (0.087)**		
**NEM effect**	(0.000)		
**PQ effect**	0 – 2.5: (0.006)	2.5 - 5: (0.002)	5 - 10: (0.850)

Table S5M. MD-BSO (2h) (see Fig. 9A)

Glutathione assessment

	Total glutathione (nmoles)			
**MD**	**-BSO**		**+BSO**	
**(mM)**	**AV**	**SD**	**AV**	**SD**
**0**	5.63	0.20	4.54	0.23
**5**	6.96	0.47	6.49	0.13
**7.5**	4.08	0.98	2.77	0.51
**10**	4.28	0.05	2.17	0.08

AV: average value. SD: standard deviation.

Statistical analysis

**Interaction**	**Does not exist (0.179)**		
**BSO effect**	(0.007)		
**MD effect**	0 – 5: (0.086)	5 – 7.5: (0.002)	7.5 - 10: (0.094)

Table S5N. MD-NEM (2h) (see Fig. 9B)

Glutathione assessment

**MD (mM)**	**Total glutathione (nmoles)**			
	**-NEM**		**+NEM**	
	**AV**	**SD**	**AV**	**SD**
**0**	3.53	2.25	2.94	0.40
**5**	4.33	0.04	2.38	0.00
**7.5**	3.60	0.18	2.75	0.32
**10**	3.62	0.15	2.72	0.35

AV: average value. SD: standard deviation.

Statistical analysis

**Interaction**	**Does not exist (0.126)**		
**NEM effect**	(0.127)		
**MD effect**	0 – 5: (0.100)	5 – 7.5: (0.003)	7.5 - 10: (0.099)

Table S5O. pH-BSO (24h) (see Fig. 9C)

Glutathione assessment

**pH**	**Total glutathione (nmoles)**			
	**-BSO**		**+BSO**	
	**AV**	**SD**	**AV**	**SD**
**7**	2.66	0.10	2.50	0.05
**5**	2.90	0.06	2.72	0.03

AV: average value. SD: standard deviation.

Statistical analysis

Interaction	Does not exist (0.730)
**BSO effect**	(0.025)
**pH effect**	pH7 – pH5: (0.001)

Table S5P. pH-NEM (24h) (see Fig. 9D)

Glutathione assessment

pH	Total glutathione (nmoles)			
	-NEM		+NEM	
	AV	SD	AV	SD
**7**	5.46	0.31	5.16	0.22
**5**	5.45	0.09	5.05	0.16

AV: average value. SD: standard deviation.

Statistical analysis

Interaction	Does not exist (0.761)
**NEM effect**	(0.006)
**pH effect**	pH7 – pH5: (0.744)

Table S6. GR structural features from selected ciliates.

Ciliate	Identifier/name	Ps (aa)	Mm (KDa)	Introns Nº	Active site CXXXXC	FAD BD GXGXXG	NADPH BD XGXXA
Tetrahymena thermophila	TTHERM_01222600 / TthGR	510	56.73	5	GGTCVNVGCVP	GGGSGG	IGPQA
Tetrahymena borealis	EI9_14674.1 / TboGR	483	53.55	4	GGTCVNVGCVP	GGGSGG	IGRNA
Tetrahymena elliotti	EI7_08658.1 / TelGR1	510	56.77	5	GGTCVNVGCVP	GGGSGG	IGRSA/VGPQA
	EI7_08658.2 / TelGR2	484	53.57	4	GGTCVNVGCVP	GGGSGG	IGRSA/VGPQA
Tetrahymena malaccensis	EIA_14085.1 / TmaGR1	483	53.39	4	GGTCVNVGCVP	GGGSGG	IGPQA
	EIA_14085.2 / TmaGR2	510	56.69	5	GGTCVNVGCVP	GGGSGG	IGPQA
Paramecium tetraurelia	PTET.51.1.P0580242 / PteGR1	475	52.08	2	GGTCPNVGCIP	GGGSGG	LGPDA
	PTET.51.1.P0890037 / PteGR2	471	52.12	2	GGTCVNVGCIP	GGGSGG	IGPEA
Stylonychia lemnae	Contig12335.g13165 / SleGR	471	51.79	0	GGTCVNVGCVP	GAGSGA	IGSHA
Stentor coeruleus	SteCoe_31278 / ScoGR1	445	49.07	0	GGTCVNLGCVP	GGGSGG	NGYIA/PGIYA
	SteCoe_33615 / ScoGR2	452	50.24	0	GGTCVNVGCVP	GGGSGG	NGYIA/VGLHA
Blepharisma stoltei	BSTOLATCC_MAC10697 / BstGR1	447	49.06	0	GGTCVNLGCVP	GGGSGG	IGRKA
	BSTOLATCC_MAC4817 / BstGR2	450	49.14	0	GGLCVNNGCVP	GGGSGG	EGLHA/IGRDA
	BSTOLATCC_MAC4818 / BstGR3	450	49.31	0	GGLCVNNGCVP	GGGSGG	GGYHA

Ps (Protein size, aa: amino acids), Mm (molecular mass), BD (Binding Domain), (X): any amino acid. Cysteine (C) residues from catalytic domains are shaded in yellow.

Tables S7 and S8. Relative fold induction values of TthGCL or TthGR gene expression under different stress conditions. Values highlighted in yellow are those that differ significantly from the control ($p < 0.05$). These values represent the average of three independent measurements and are shown in Fig. 13 or Fig. 14 histograms.

Table S7.

Treatment	Exposure time (h)	TthGCL gene Fold induction	TthGCL gene SD	TthGCL gene P value
**Cd(II)**	2	7	2.89	0.033
	24	4	1.87	0.795
**Cu(II)**	2	2	0.76	0.824
	24	0.85	0.09	0.663
**As(V)**	2	89	36.0	0.001
	24	4	1.20	0.032
**Pb(II)**	2	6.5	1.90	0.026
	24	5	1.45	0.088
**Zn(II)**	2	7	3.04	0.036
	24	0.85	0.05	0.594
**PQ**	24	6	3.16	0.016
**MD**	2	123	31.0	0.017
**CDNB**	2	13	2.0	0.001
**pH 5**	24	120	44.0	0.001
**pH 9**	24	0.77	0.30	0.109
**Starvation**	24	3	0.55	0.175

SD: Standard deviation

Table S8.

Treatment	Exposure time (h)	TthGR gene Fold induction	TthGR gene SD	TthGR gene P value
**Cd(II)**	2	0.74	0.57	0.676
	24	5.16	0.80	0.001
**Cu(II)**	2	1.81	0.85	0.514
	24	0.65	0.66	0.845
**As(V)**	2	205.07	80.81	0.001
	24	107.73	23.39	0.001
**Pb(II)**	2	42.58	6.11	0.018
	24	43.39	5.78	0.015
**Zn(II)**	2	0.40	0.15	0.167
	24	0.17	0.02	0.175
**PQ**	24	0.48	0.06	0.175
**MD**	2	0.84	0.28	0.833
**CDNB**	2	111.38	40.78	0.001
**pH 5**	24	0.71	0.11	0.741
**pH 9**	24	0.02 ⬅	0.03	0.001
**Starvation**	24	0.24	0.04	0.151

SD: Standard deviation. (⬅): Although there is a significant difference, it is not considered a real induction as it is < 2-fold.

Text S1.

To make it easier to understand the tables on the statistical analyses used to determine -SH groups, total glutathione, and cell mortality, the following examples illustrate the different types of results that could be obtained.

We first examined the possibility of interaction between the two factors used in this study: the inhibitors (BSO or NEM) and the stressors (Cd(II), As(V), PQ, MD, or pH 5).

Table S9. No interaction.

**Interaction**	**Does not exist (0.360)**		
**BSO effect**	(0.364)		
**Cd(II) effect**	0 – 1.32 (0.763)	1.32 – 1.54 (0.708)	1.54 – 1.76 (0.539)

Since the p-value is 0.360, which is greater than 0.05, there is no interaction between Cd(II) and BSO; in other words, the behavior of Cd(II) does not affect the behavior of BSO, and vice versa.

When there is no interaction, the effects of the two factors are analyzed jointly across the entire sample. The effect of BSO is not statistically significant (0.364 > 0.05). Similarly, the effect of Cd(II) is also not statistically significant across the three concentration ranges used, since 0.763, 0.708, and 0.539 are all > 0.05 when analyzed separately (Table S9).

On the other hand, there may be cases in which there is an interaction between the two factors: Cd(II)-induced stress and BSO-mediated GSH inhibition. As shown in the example below.

Table S10. Interaction.

**Interaction**	**Exists (0.000)**		
**BSO effect**			
**Cd(II) (0): (0.000)**	Cd(II) (1.32): (0.001)	Cd(II) (1.54): (0.002)	Cd(II) (1.76): (0.000)
**Cd(II) effect**			
**BSO (-)**	0 - 1.32: (0.004)	1.32 - 1.54: (0.013)	1.54 – 1.76: (0.048)
**BSO (+)**	0 – 1.32: (0.001)	1.32 – 1.54: (0.090)	1.54 - 1.76: (0.539)

In the example shown in Table S10 an interaction exists, since the p-value is less than 0.05 (values shaded in yellow). The presence of an interaction means that Cd(II) behaves differently in the presence or absence of BSO, and vice versa.

When there is an interaction between the two factors, the analysis must be different. The BSO or NEM factors have only two levels of influence: presence or absence. To analyze their effect, a comparison of means of independent samples is performed for each concentration of the other factor (stressor). In our example, the effect of BSO is analyzed for each of the Cd(II) concentration ranges used. Each stressor has four levels (control and three different concentrations).

If an interaction between the toxicant and the inhibitor is detected, the results must be analyzed separately: on the one hand, treatments conducted without BSO or NEM, and on the other hand, those conducted in its presence.

In samples without BSO, there are significant differences across all concentrations of Cd(II) used (Table S10, values highlighted in yellow). However, in samples exposed to BSO, Cd(II) has a significant effect only at the first two concentrations (0 and 1.32 μM Cd(II)).
